# On the pathway of cellular uptake: new insight into the interaction between the cell membrane and very small nanoparticles

**DOI:** 10.3762/bjnano.7.121

**Published:** 2016-09-16

**Authors:** Claudia Messerschmidt, Daniel Hofmann, Anja Kroeger, Katharina Landfester, Volker Mailänder, Ingo Lieberwirth

**Affiliations:** 1 Max Planck Institute for Polymer Research, Ackermannweg 10, 55128 Mainz, Germany; 2 Ostwestfalen-Lippe University of Applied Sciences, Liebigstr. 87, 32657 Lemgo, Germany; 3 Dept. of Medicine III, Hematology, Oncology and Pneumology, University Medical Center of the Johannes Gutenberg University Mainz, Langenbeckstr. 1, 55101 Mainz, Germany

**Keywords:** ATP depletion, calcium crystallization, cytotoxicity, endocytosis, HeLa cells, LDH, mesenchymal stem cells, morphology, necrosis, particle size, silica nanoparticles, TEM

## Abstract

For any living cell the exchange with its environment is vital. Therefore, many different kinds of cargo are able to enter cells via energy-dependent or -independent routes. Nanoparticles are no exemption. It is known that small silica nanoparticles with a diameter below 50 nm are taken up by cells and that their uptake exerts pronounced toxic effects beyond a certain concentration threshold. However, neither the exact uptake mechanism of these particles nor the actual reason for their toxicity has yet been elucidated. In this study we examined the uptake of silica nanoparticles with a diameter of 7, 12 and 22 nm by means of transmission electron microscopy, accompanied by toxicological assays. We show that for every particle diameter tested a different membrane morphology during uptake can be observed and that the amount of particles entering in one event is different for the three sizes. Silica particles with a diameter of 22 nm show single-particle internalization with a membrane wrapped around the particles in the cytosol, whereas 12 nm particles display row-like multi-particle uptake into elongated membrane structures and those with a diameter of 7 nm or less end up in tubular endocytic structures containing many particles. These membrane morphologies proved to be highly reproducible as we found them in five different cell lines. Additionally, we performed ATP and LDH assays to determine particle toxicity. Exceeding a certain concentration threshold the nanoparticles showed a high toxic potential both in the biochemical assay measurements and from morphological findings. We could not find any hint at the induction of apoptosis, neither morphologically nor biochemically. In this regard we discuss membrane damage and consumption as one possible mechanism of toxicity, linking morphological observations to toxicological findings to bridge the gap in understanding the mechanism of toxicity of small nanoparticles.

## Introduction

Silicon dioxide nanoparticles (SiNPs) are used in a wide range of commercially available products to improve product features. As free-flow or anti-caking agent they are contained, e.g., in powdery products of the food industry, serve as fragrance carriers in cosmetics or as the abrasive component in toothpaste. This results in an exposure to SiNPs on a more or less regular basis. Though no acute adverse effects of SiNPs have been reported in humans, general risk assessment and thereby the investigation of possible interactions of SiNPs with human cells and tissues is of crucial importance. A deeper understanding of SiNP uptake modes into cells may lead us one step further in grasping nanoparticle cytotoxicity and in paving the way for novel biotechnological and medical applications.

Generally, in vitro studies report that SiNPs are taken up by cultured human cells. Nevertheless, the exact mechanism of this process still needs to be elucidated in detail for a better understanding of the cytotoxic effects or to develop tailored particles that are perfectly suitable for specific applications in the life sciences. Many groups conclude that this uptake happens through active energy-dependent processes and follows the usual endolysosomal pathway [1–6]. Using different microscopic and immunological methods, the respective endolysosomal vesicles frequently have been shown to contain SiNPs, whereas the cell nucleus is only occasionally reported to have engulfed nanoparticles [5,7]. On the contrary, there are observations corroborating the hypothesis of the SiNPs’ ability to passively pass through lipid membranes and enter cells [8–10]. For example, Mu et al. found uptake of SiNPs (14 nm diameter) even in cells that were kept at 4 °C, a temperature at which active processes are conceived to be significantly suppressed [9]. In subsequent TEM analysis they observed particles freely in the cytosol without any membrane wrapping. In addition, Lesniak et al. report free SiNPs (50 nm diameter) in the cytosol after treatment with higher particle concentrations [8]. Furthermore, it has been repeatedly measured by different groups that the number of particles entering a cell in a given time is dependent on particle size and the serum content in the culture medium [11–12]. In serum-free conditions the uptake of SiNPs has been determined to be much higher than in the presence of fetal calf serum (FCS), which is attributed to the fact that SiNPs build up a protein corona and tend to agglomerate in serum-containing media [8,11,13–14]. Interestingly, studies using cell models like artificial liposomes or polymersomes report the uptake of SiNPs into these structures in a size-dependent manner [15].

Different groups report that silica NPs are cytotoxic in a dose, size and time-dependent manner. Mostly, it is assumed that the smaller a nanoparticle, the more pronounced its toxic effect [7,16–22]. Furthermore, the mode of cell death occurring is usually believed to be necrosis, but some groups have found that the apoptotic machinery is activated [23–26]. In addition to that, SiNPs seem to have the potential of disturbing Ca^2+^ homeostasis [27].

The aim of our study is to expand the window of nanoparticle–cell membrane ultrastructural investigations to particle sizes well below 25 nm in diameter. Little electron microscopic information exists in this size regime. As model cell systems we chose different epithelial and non-epithelial cell lines of carcinoma and primary origin which were exposed to silica NPs. This variety of model cell lines was deliberately selected to check for the universality of our observations. Using electron microscopic methods, we aim at watching if and how such small silica particles enter the cytosol and if the morphology of the uptake process reveals a clue to the cause of NP toxicity. In order to increase the probability of catching the very moment of uptake by means of TEM, it is either necessary to inspect the process shortly after the incubation or to work with non-physiological high particle concentrations. The latter approach caused high cell lethality after only a few hours of incubation. Accordingly, we conducted additional toxicity tests in order to quantitatively correlate the observed morphology to the cytotoxic potential of the applied silica NPs.

In this study we used electron microscopic (EM) methods together with high pressure freezing preparation to investigate the morphological details of silica NP uptake into cultured cells. Yielding a good preservation of ultrastructure, these methods enabled us to have a detailed look at the very moment of particle uptake, revealing a remarkable dependency of the observed morphologies on particle size. Alongside the EM analysis we applied confocal laser scanning microscopy (CLSM), biochemical assays for LDH and ATP, flow cytometry, Caspase-3 western blotting and Hoechst staining to collect data about the cytotoxic effects already indicated by EM.

## Results

### Nanoparticle characterization

A prerequisite for detailed studies of cell–NP interaction is a thorough characterization of the applied particles. Therefore we determined essential particle properties using transmission electron microscopy (TEM) and dynamic light scattering (DLS), especially under consideration of the application conditions in physiological media. The three differently sized silica NPs investigated throughout this study, were used as purchased without further surface modification. The particle diameters specified by the manufacturer were 7 nm, 12 nm and 22 nm and will be referred to as SiNP-7, SiNP-12 and SiNP-22, respectively.

**Figure 1:**
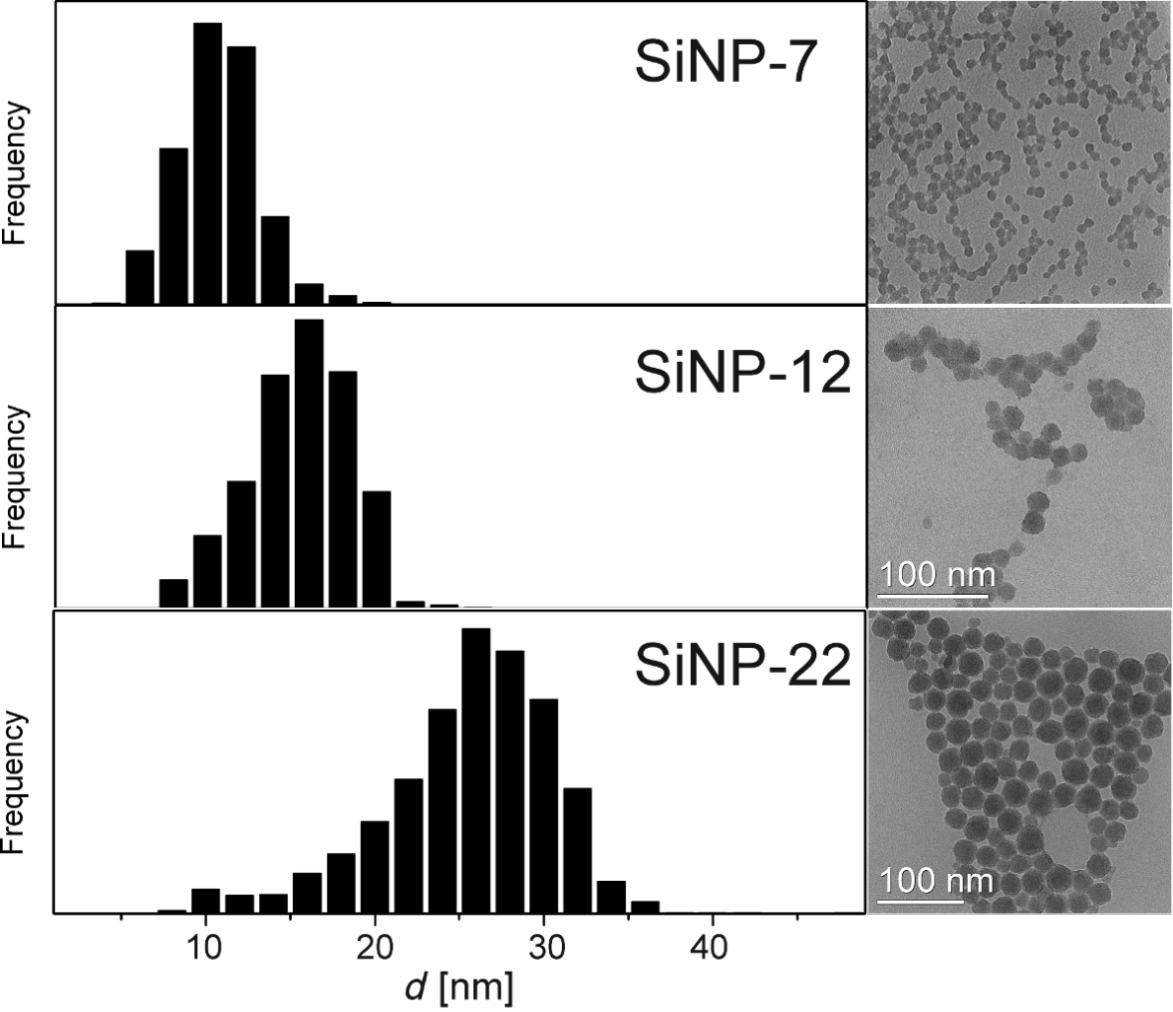
Microscopic *d*etermination of NP size from TEM image analysis.

Figure 1 shows the measured size distributions and representative TEM bright field micrographs of the respective SiNPs. Table 1 gives the average radius *r_n_* and the *z*-weighted radius *r_z_* from the entirety of measured particles with


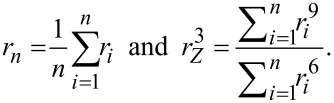


**Table 1:** Average radius and ζ-potential of the SiNPs as determined by TEM, DLS and electrophoretic mobility measurements, respectively. TEM statistics are based on the measurement of at least 1500 individual nanoparticles.

trade name	sample name	*r_n_* (TEM) [nm]	*r_Z_* (TEM) [nm]	*R*_H_ (DLS) [nm]	PDI (DLS)	ζ-potential [mV]

Ludox TMA	SiNP-22	12 ± 3	15 ± 3	19 ± 2	0.14	−31.6
Ludox HS-30	SiNP-12	7 ± 2	9 ± 2	10 ± 1	0.17	−11.2
Ludox SM-30	SiNP-7	5 ± 1	7 ± 1	11 ± 1	0.26	−25.3

The values for the effective *z*-average Stokes–Einstein hydrodynamic radii *R*_H_ as well as the polydispersity index PDI (determined by the Cumulant method) are given in Supporting Information File 1, Table S1. In order to compare the TEM and DLS results, the averages from the TEM measurements are given as *z*-averages as well. For SiNP-22 and SiNP-12 the TEM and DLS measurements are in good agreement. For SiNP-7 however, the TEM and DLS values seem to be inconsistent at first sight. For this sample a much higher PDI was determined. However, systematic errors of TEM micrograph processing together with a broad size distribution may be causal for this discrepancy.

Since DLS measurements of nanoparticle size distribuitons are carried out in solution, the hydration shell of the nanoparticles additionally contributes to the particle radius value. On the other hand, TEM measurements require dried samples and hence no additional hydration shell contributes. Accordingly, DLS will systematically yield a slightly larger average particle size than the TEM measurement. Furthermore, DLS measurement integrates, as every scattering measurement, over a large ensemble whereas microscopic studies can only account for a very limited number of individual objects, resulting in a poor statistics of imaging-based size measurements. Additionally, in DLS measurements the presence of a minor population of associated particles cannot be excluded although the measurements were done at very low concentration. This might additionally elevate the average size.

The particle surface was characterized with regards to its ζ-potential. The surface of all three applied SiNPs was negatively charged (data shown in Table 1). According to the manufacturers data sheet the particle surface is terminated with OH-groups.

**Figure 2:**
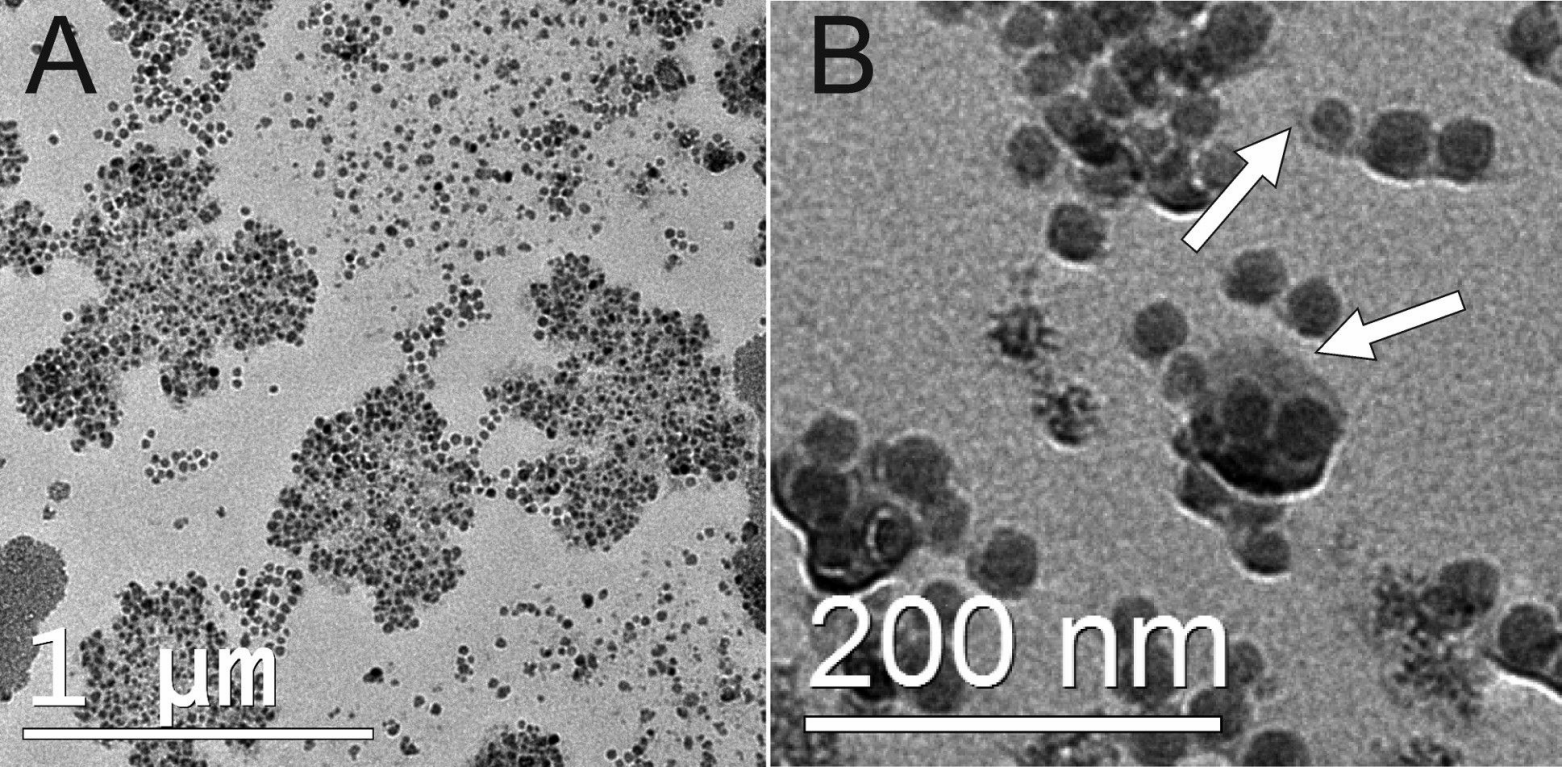
TEM bright field micrograph of SiNP-22 in the presence of DMEM and FCS showing large aggregates of SiNPs (A). Closer inspection of the silica particles even displays their coverage with a presumably organic / protein layer (arrows in B).

In addition to water, the SiNPs were measured in DMEM and DMEM+FCS containing media as well (Table 2). For DMEM media, no significant changes in the hydrodynamic radii were observed, but when adding FCS, all the three different SiNPs form large aggregates. Figure 2 exemplarily shows aggregates of SiNP-22 as seen by TEM after drying from DMEM+FCS containing medium. On closer inspection of the micrographs one can even recognize an adsorption layer around the SiNPs (Figure 2B). Hence, we might attribute the immediate agglomeration of SiNPs to the adsorption of proteins to the silica surface followed by some kind of flocculation process. The hydrodynamic radius of these agglomerates was in the order of 100 to 300 nm (Table 2, Figure 2A).

**Table 2:** Hydrodynamic radii of SiNPs in water, DMEM and DMEM + FCS.

	*R*_H_ in H_2_O [nm]	*R*_H_ in DMEM [nm]	*R*_H_ in DMEM+FCS [nm]

SiNP-22	19 ± 2	17 ± 2	112 ± 20
SiNP-12	10 ± 1	11 ± 1	237 ± 20
SiNP-7	11 ± 1	12 ± 1	197 ± 20

### Morphological examination of the NP–cell membrane interaction

**Figure 3:**
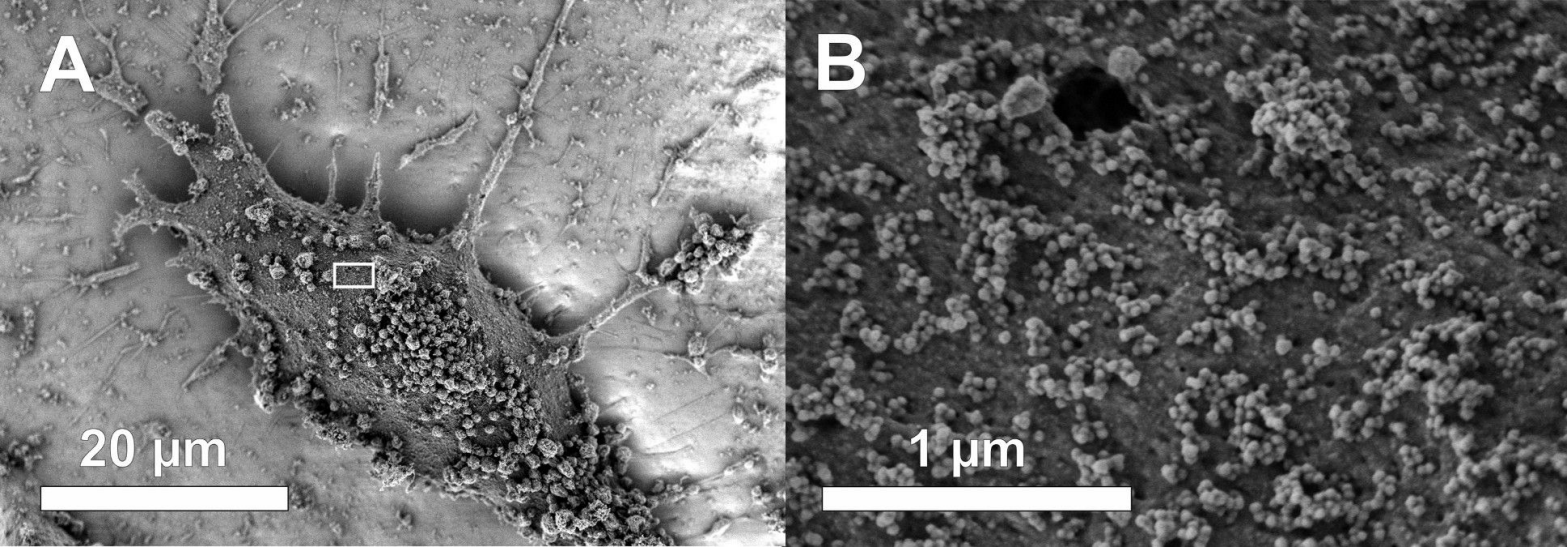
SEM micrograph of a HeLa cell exposed to 100 µg·mL^−1^ of SiNP-22 for 15 min before CPD preparation. Besides the large agglomerates the surface of the cell is covered with many individual, non-aggregated NPs (B, close-up of the area indicated in A).

This directly raises the question, if the agglomeration of NPs plays a crucial role in their uptake into cells. Accordingly, one has to examine the adsorption of the NP agglomerates on the cell membrane. Figure 3 shows a scanning electron microscope (SEM) micrograph of a HeLa cell exposed to 100 µg·mL^−1^ SiNP-22 for 15 min prior to fixation. It is worth mentioning that during the preparation (by an alcohol dilution series followed by critical point drying (CPD)) the sample has been washed several times and hence only the firmly attached NPs stayed on the cell membrane. The coverage of the cell membrane consists of individuals, small groups and large agglomerates of NPs. Despite the tendency of the NPs to build up agglomerates in serum-containing medium, we therefore conclude from the SEM micrographs, that individual NPs still play an important role in uptake processes.

**Figure 4:**
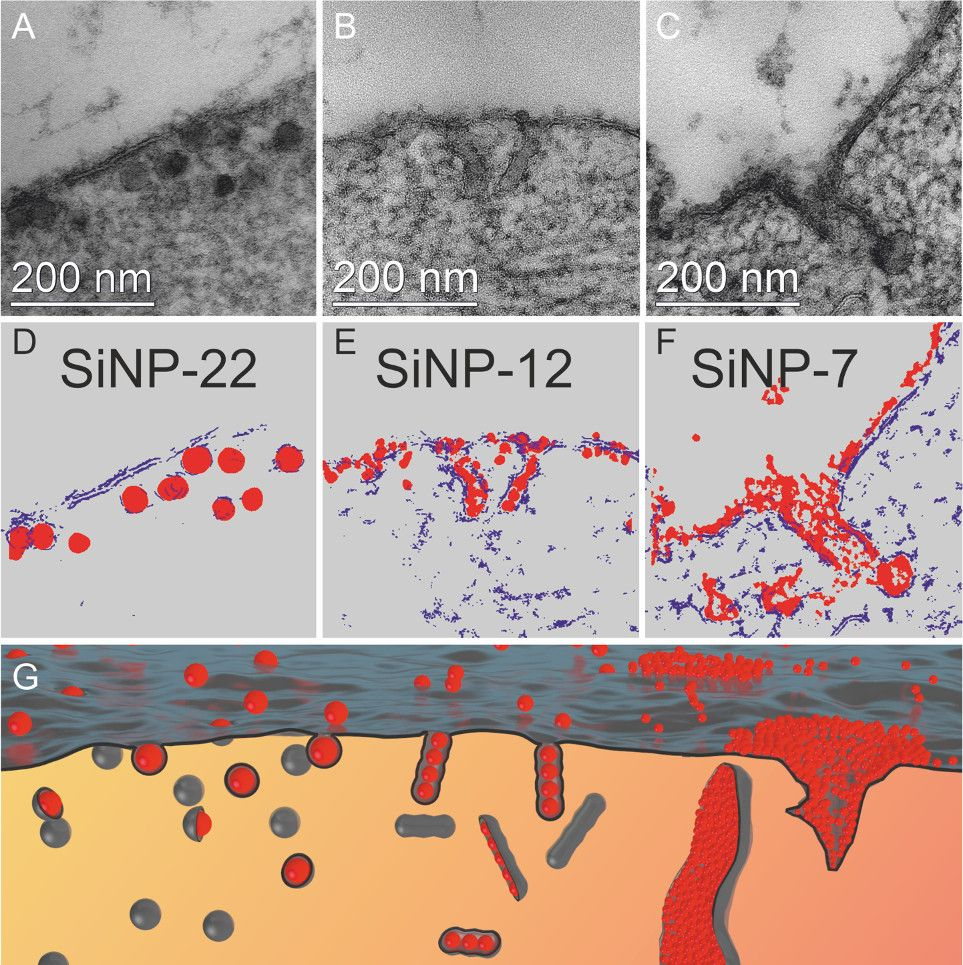
TEM brightfiled micrographs of high-pressure frozen HeLa cells exposed to SiNPs at a concentration of *c* = 75 µg·mL^−1^ for 10 min, stained with OsO_4_ and uranyl acetate (micrographs A–C show SiNP-22, SiNP-12 and SiNP-7, respectively). For clarification purpose the illustrations in D,E represent the membrane (blue) and the SiNPs (red) as extracted from energy filtered TEM measurements of the above micrographs. Schematic representation of the membrane–particle interaction morphology (G) observed for the different particle sizes.

**Figure 5:**
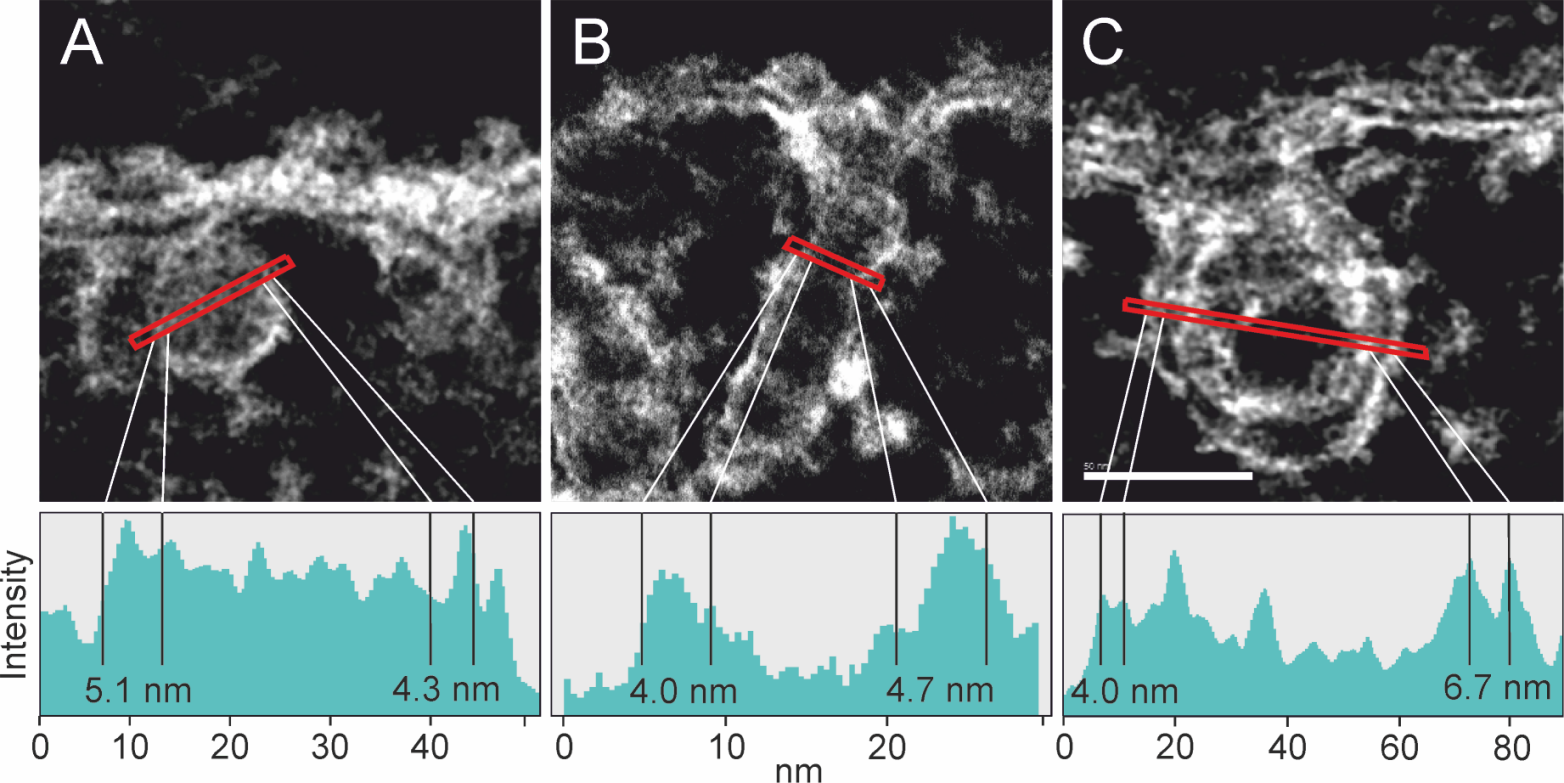
STEM micrographs showing the particle surrounding membrane formed upon uptake of SiNP-22 (A), SiNP-12 (B) and SiNP-7 (C), respectively. The thickness of the membrane is measured from the intensity profile (below). Membrane thickness varies little and is found to be between 4 and 7 nm for all three different particle sizes. For SiNP-22 and SiNP-12 the typical contrast characteristic for a double layer membrane is unincisive as can be seen in the respective intensity profiles. Experimental conditions same as in Figure 4. Scale bar = 50 nm.

This is corroborated by the TEM examination of the corresponding ultrastructure of the NP–cell membrane interaction. When looking at the ultrastructure, we actually discovered three different uptake morphologies depending on the NPs’ size. SiNP-22 particles penetrate the membrane as individuals (Figure 4A,D). Per uptake event, one individual particle passes the membrane. Moreover, Figure 4A displays an uptake event at the very moment the particle enters the cell. (However, since TEM imaging is a static measurement, we only can assume the direction of this event. Due to the short incubation time we favor the endocytotic over the exocytotic process.) One can observe that the silica particle is enclosed by a membrane but still connected to the outer cell membrane. The intensity profiles of the individual surrounded particles in scanning transmission electron microscope (STEM) micrographs are clearly indicative of a lipid membrane as the measured thickness of the surrounding membrane is approximately 4 nm (Figure 5A,D). This roughly is the thickness of two lipid tails in a phospholipid membrane. The distinct contrast in the STEM micrograph is attributed to the existence of C–C double bonds in the bilayer structure, which are stained by OsO_4_. Furthermore, micrographs show agglomerated particles outside the cell whereas the uptake seems to proceed via single particle events (Supporting Information File 1, Figure S2 and Figure S5). Rarely, we discovered a pair of particles sharing one vesicular membrane.

SiNP-12 shows a completely different ultrastructural uptake morphology. The uptake process involves several particles at a time. In average 5 to some tens of NPs participate per uptake event. In the cytosol they are lined up in a row and wrapped in a membrane (Figure 4B and Supporting Information File 1, Figure S3).

For SiNP-7 a further change of uptake morphology is observed (Figure 4C, Supporting Information File 1, Figure S7 and Figure S9). Large amounts of NPs cover the membrane and in certain areas form a tubular invagination into the cell. Additionally, endosome-like, ill-shaped structures filled with SiNPs are found in the cytosol (Supporting Information File 1, Figure S7).

The schematic illustration in Figure 4G summarizes the above described findings. SiNP-22, which were the largest examined particles in our study, enter the cell as individual particles. Upon transit through the outer cell membrane they receive a tightly wrapped membrane. With decreasing particle size, the uptake morphology undergoes considerable changes. Medium-sized SiNPs (SiNP-12) already do not enter the cell as individuals but in small groups which are delimited by a tight membrane as well. Significantly, these groups arrange in a row-like manner. The smallest SiNPs under examination finally induce tubular structures, lined with silica particles. These structures as well are able to constrict themselves from the membrane. These different membrane shell morphologies are quite remarkable, considering the relatively slight difference in particle size from 24 to 10 nm in diameter (as determined by TEM).

When exceeding a certain concentration, silica NPs become cytotoxic [28–30]. In order to explore, if there is a certain correlation between toxicity and the observed uptake morphology, which obviously consumes part of the outer cell membrane, we performed additional cytotoxic measurements. Preliminary, a qualitative examination of the cytotoxic effects was conducted by fluorescence activated cell sorting using FSC/SSC analysis (data not shown) and confocal laser scanning microscopy (Supporting Information File 1, Figure S10). Further experiments examined the LDH release and the adenosine-triphosphate (ATP) level of HeLa cells upon exposure to the respective SiNPs. Moreover, the cleavage of Caspase-3 was measured in Western blot experiments to investigate the activation of the major pro-apoptotic protease (Supporting Information File 1, Figure S11).

**Figure 6:**
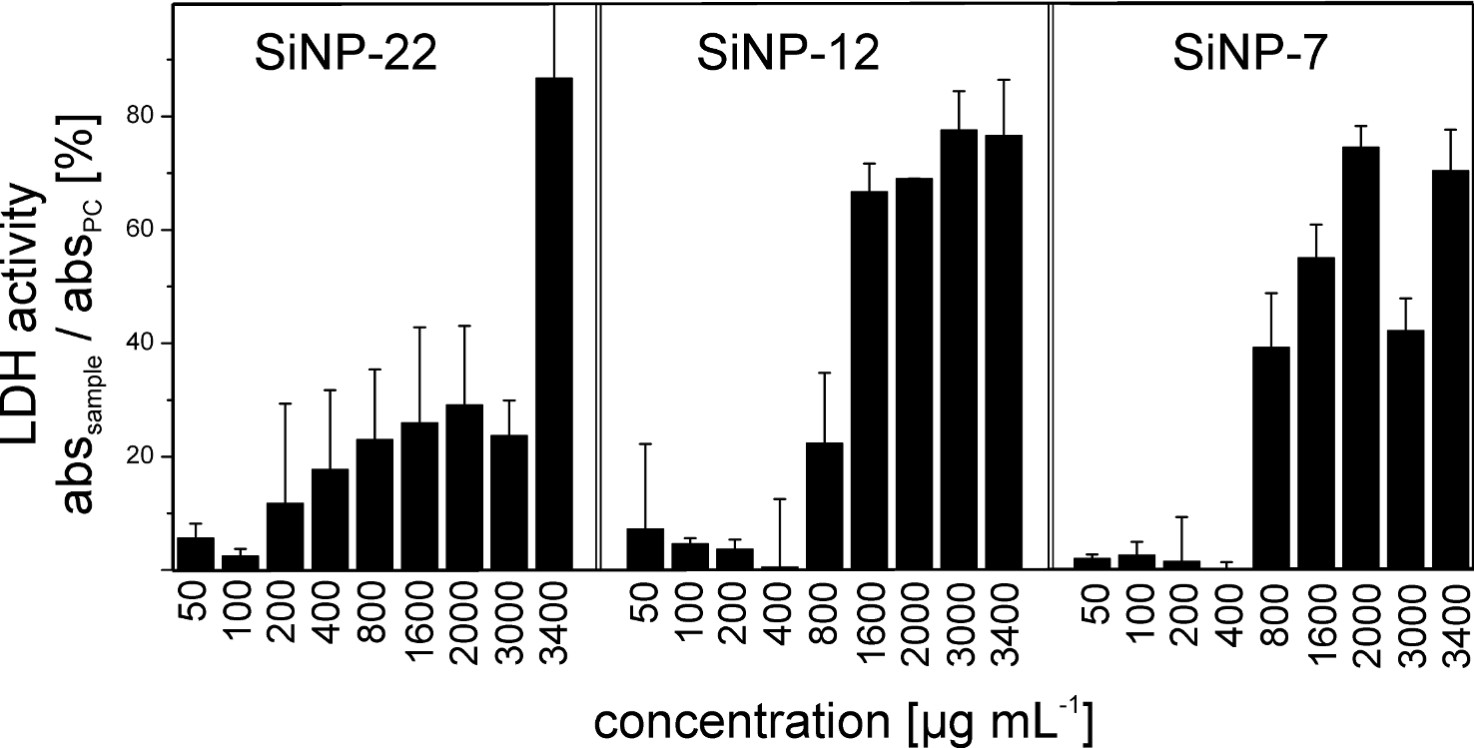
Level of LDH release of HeLa cells after incubation with silica NPs for 2 h. The extent of extracellular LDH is expressed as a normalized quotient to the positive control (PC).

The level of LDH release is indicative for the disintegration of the cell membrane and consequently for cytotoxicity. For quantitative estimation of the toxic potential HeLa cells were incubated with NPs for 2 h at different concentrations followed by determination of the LDH release (Figure 6). SiNP-22 induces only a moderate increase of the LDH level for all tested concentrations except the highest at 3400 µg·mL^−1^, where it raised to a level of nearly 90% compared to the positive control. For SiNP-12 and SiNP-7 the results are similar, up to a concentration of 400 µg·mL^−1^ the LDH levels are below 20% and with increasing concentration they raise to 50% and above. These data reveal that the smaller NPs (SiNP-12 and SiNP-7) induce cell membrane damage already at lower concentrations compared to the large NPs. However, at very high concentrations, all NPs cause substantial cell membrane disintegration.

**Figure 7:**
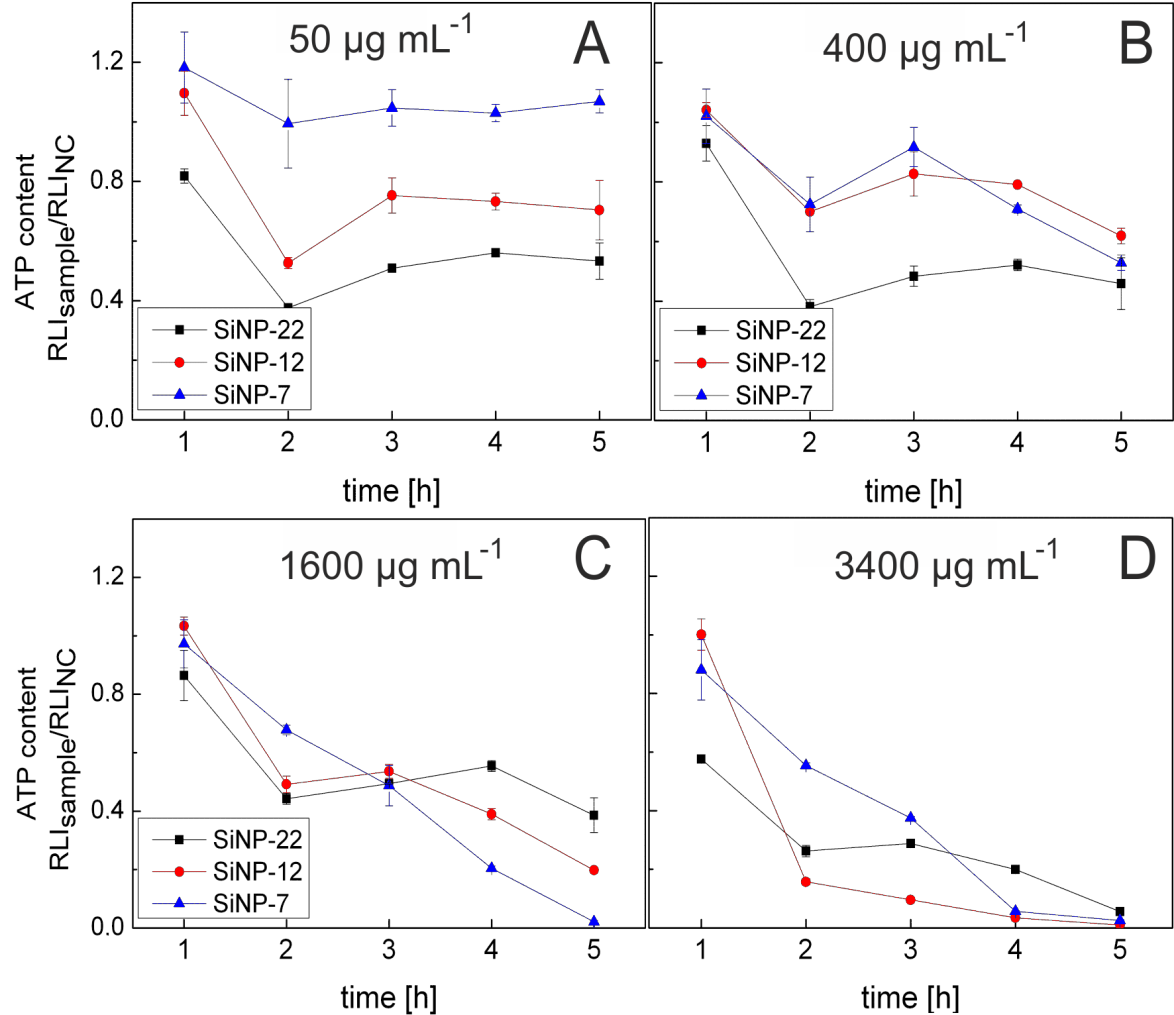
Measurement of intracellular ATP depletion. HeLa cells were exposed to SiO_2_ NPs at different NP concentrations as indicated. The extent of extracellular ATP is expressed as a normalized quotient to the negative control.

Further on, the metabolic activity of the cells was measured in response to NP exposure. The level of ATP inside a cell represents the metabolic activity mediated by the ATP synthase and its decrease is a reliable indicator for mitochondrial malfunction or starvation of the cells. Here, we measured the ATP content in dependence of NP concentration and size over 5 h of exposure in HeLa cells. Figure 7 displays the ATP level of HeLa cells exposed to NPs at 4 different concentrations and measured at time intervals of 60 min. At the lowest NP concentration (50 µg·mL^−1^, Figure 7A) SiNP-22 induces the highest ATP depletion effect, whereas SiNP-7 even shows a slight increase of ATP compared to the negative control (NC). At increased NP concentrations the ATP level decreased distinctly for all NP sizes (Figure 7B–D). From these results it is quite difficult to rank the NP regarding their cytotoxicity. One might conclude that at the lower concentrations SiNP-22 are most toxic whereas at increased concentrations this is the case for the smaller NPs (SiNP-12 and SiNP-7). Inducing of cytotoxic effect by DMSO 10% as positive control yields typically an ATP content of 10% after 48 h we find here that high concentrations of silica NPs even let the ATP content drop to values of some percent and therefore below these positive controls.

Though it is not possible to draw final conclusions concerning the mechanisms inducing cell death from the morphological data alone, it is a plausible thought that at high concentrations the massive interaction of SiNPs with cellular membranes and membrane proteins severely influences cell function and integrity.

**Figure 8:**
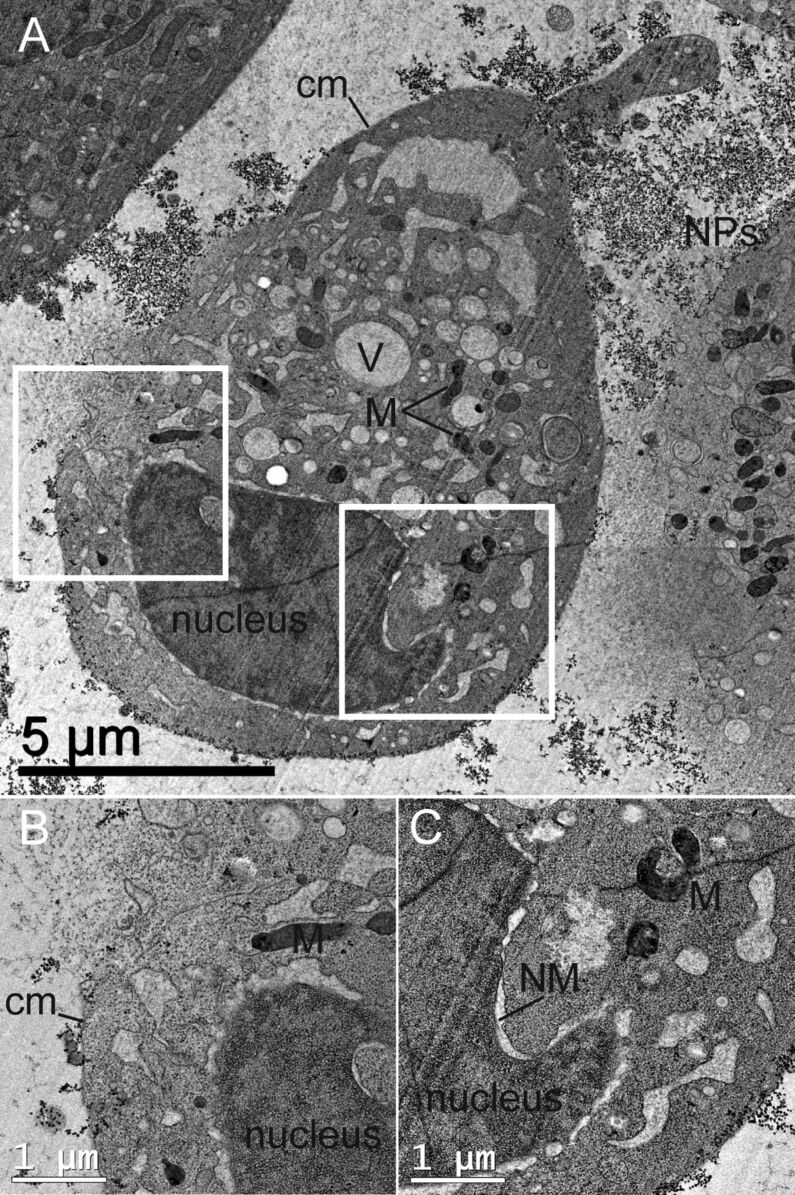
TEM micrographs of high-pressure frozen HeLa cells treated with 3400 µg·mL^−1^ of Si-NP22 for 2 h. B and C are close-ups of the central cell in A, showing the cell membrane (cm) leakage and the nuclear membrane (NM) disintegration in more detail. Additionally labeled in the micrograph are the mitochondria (M) and the vacuoles (V).

**Figure 9:**
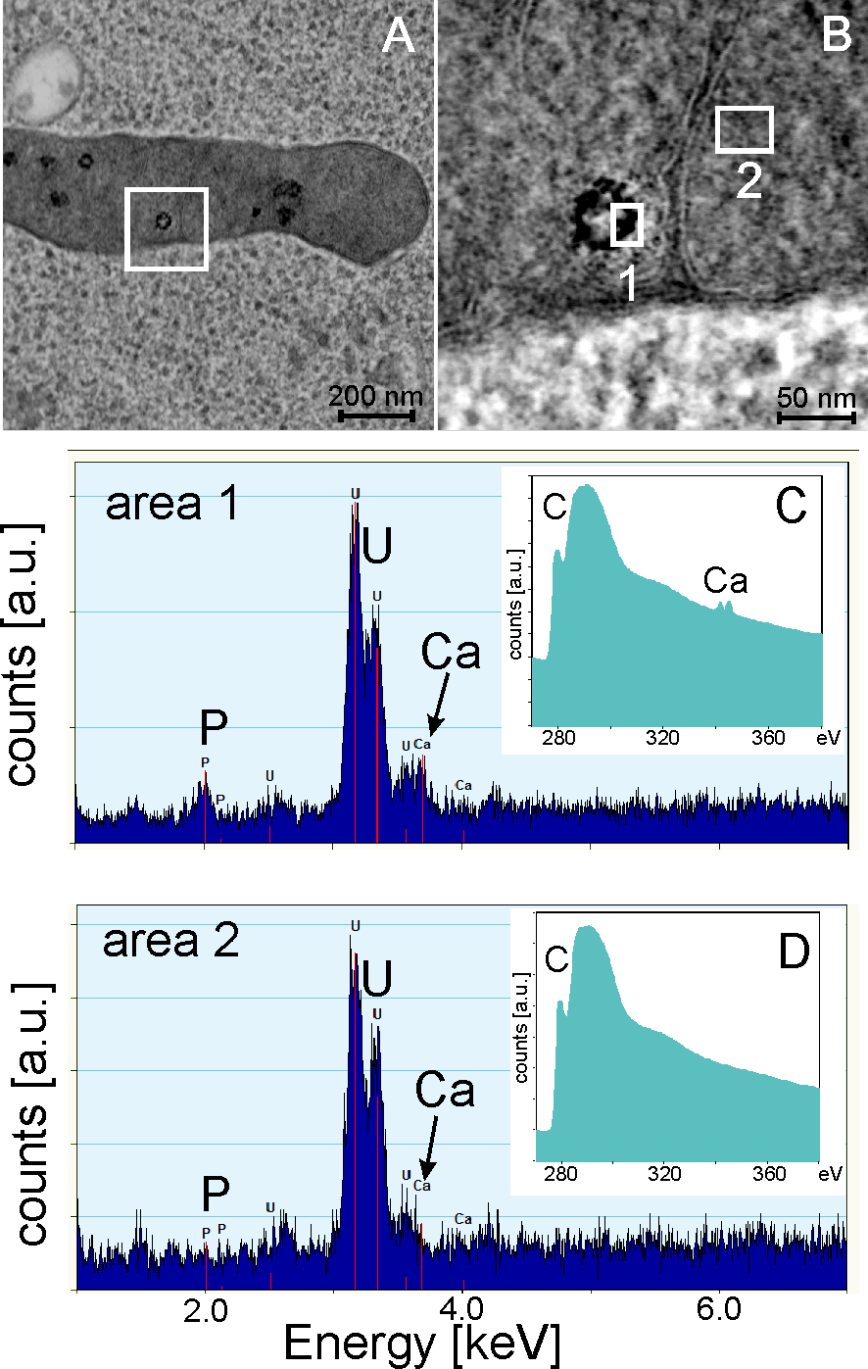
STEM high angular annular dark field (HAADF) micrograph of a mitochondrion containing darkly stained agglomerates (A) and magnification of the indicated area (B). Image contrast has been inverted to adapt the micrograph to the appearance of conventional TEM bright field images. Local EDX and EELS analysis (C and D) of the areas indicated in B.

Hence, we exemplarily incubated HeLa cells for as long as 24 h using high particle concentrations of 3400 µg·mL^−1^. The subsequent TEM examination showed only cell fragments for all three particle sizes. Qualitative FACS measurements revealed that all HeLa cells being exposed to these concentrations are dead after only 5 h, indicating strong toxic effects (data not shown). For SiNP-12 and SiNP-22, even after only 2 h of incubation we found a significant number of cells with a leakage in the outer membrane and general signs of necrosis like vacuolization, nuclear membrane disintegration and mitochondria containing agglomerates of dark appearance in TEM brightfield micrographs (Figure 8). From the morphological point of view, the size and frequency of these agglomerates correlates with the overall appearance of the cell – the more advanced the necrotic cellular breakdown the more distinct the dark material inside the mitochondria. These agglomerates might be due to deposition of Ca^2+^ salts within the mitochondria. EELS and EDX measurements revealed a significantly raised content of calcium in these intramitochondrial agglomerates compared to the mitochondrial matrix and the surrounding cytoplasm (Figure 9).

Generally, we observed that all SiNPs under examination exert toxic effects when exceeding a certain concentration. Evaluating the overall morphological observations, we found many hints at necrotic cell death involving membrane disintegration, cell swelling (and even bursting, Supporting Information File 1, Figure S12), vacuolisation and Ca^2+^-containing agglomerates inside mitochondria. In EM and cLSM experiments we could not observe any apoptotic bodies. After longer incubation times we only detected diffuse cell debris in EM. Additionally, we could not detect Caspase-3 cleavage, a central event in extrinsic and intrinsic apoptosis. The increase in LDH leakage is another hint at elevated membrane damage and necrosis. However, it is not the scope of our study to look for possible activations of the apoptotic machinery in depth.

**Figure 10:**
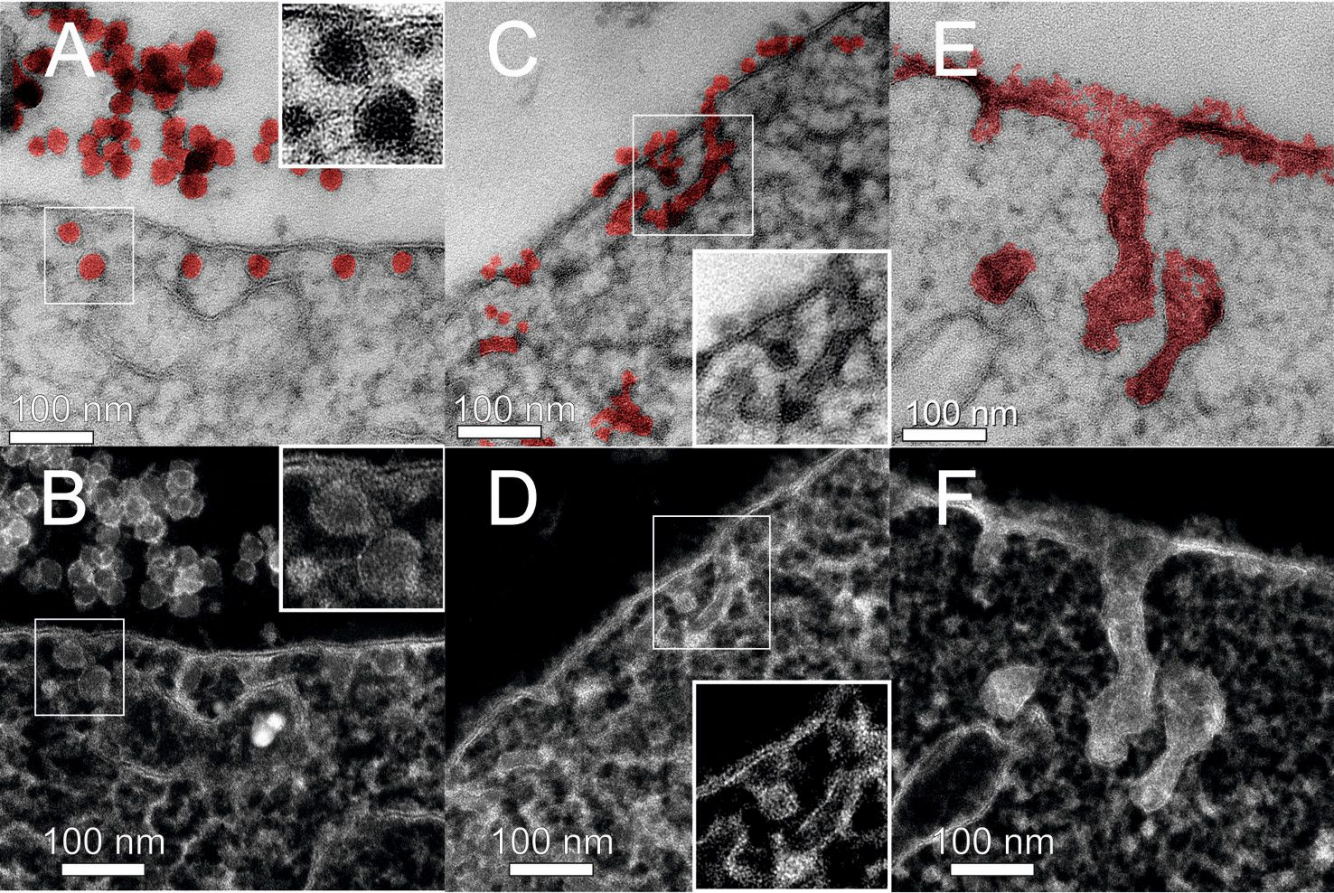
TEM micrographs of high-pressure frozen HeLa cells treated with high concentrations of SiNPs for 2 h, stained with OsO_4_ and uranyl acetate. Particle concentrations are *c* = 3400 µg·mL^−1^ for SiNP-22 (A,B), *c* = 3000 µg·mL^−1^ for SiNP-12 (C,D) and *c* = 3000 µg·mL^−1^ (E,F) for SiNP-7. The upper images are bright field micrographs using a pseudocolor for Si containing areas as extracted from energy filtered TEM micrographs. The bottom row (B,D,F) displays the corresponding STEM micrographs with enhanced contrast, clarifying the particle enclosing membranes.

However, even at non-physiological high concentrations of 3400 µg·mL^−1^ we could observe the same size dependent morphological features for the particle uptake which we already saw at low concentrations (Figure 10). Again, SiNP-22 are taken up as individuals, SiNP-12 in a row like manner and SiNP-7 induce tubular invaginations and all these structures were tightly enclosed by a membrane as well.

The above described differences in membrane morphology during uptake were rather surprising and unexpected and raise the question, if this observation is limited to HeLa cells only or if this is a universal mechanism with which a cell and its membrane will react upon treatment with small silica NPs. Accordingly, we tested another 4 cell lines for the uptake morphologies: primary human mesenchymal stem cells (hMSC), human bone osteosarcoma cells (U2OS), human epithelial colorectal adenocarcinoma cells (Caco-2) and mouse melanoma (B16-F10) cells. Caco-2 cells are very often used as model of the human intestinal barrier. Furthermore we investigated HeLa and U2OS cells. These cell lines were not derived from directly relevant tissues but can serve as epithelial models as well. hMSCs were used because of their primary origin, to investigate if similar morphologies are observable in primary, non-malignant cells. To further check for universality, we finally exposed the mouse cell line B16-F10 to SiNPs. For all these cell lines we observed similar uptake morphologies; single particle uptake for SiNP-22, row-like morphologies for the medium sized SiNP-12 and tubular uptake structures for the smallest SiNP-7 particles (Supporting Information File 1, Figure S5, Figure S6, Figure S7).

This morphological consistency between different cell lines and NP concentrations is remarkable. Accordingly, we can look for the uptake microstructure using any of the above mentioned cell lines.

**Figure 11:**
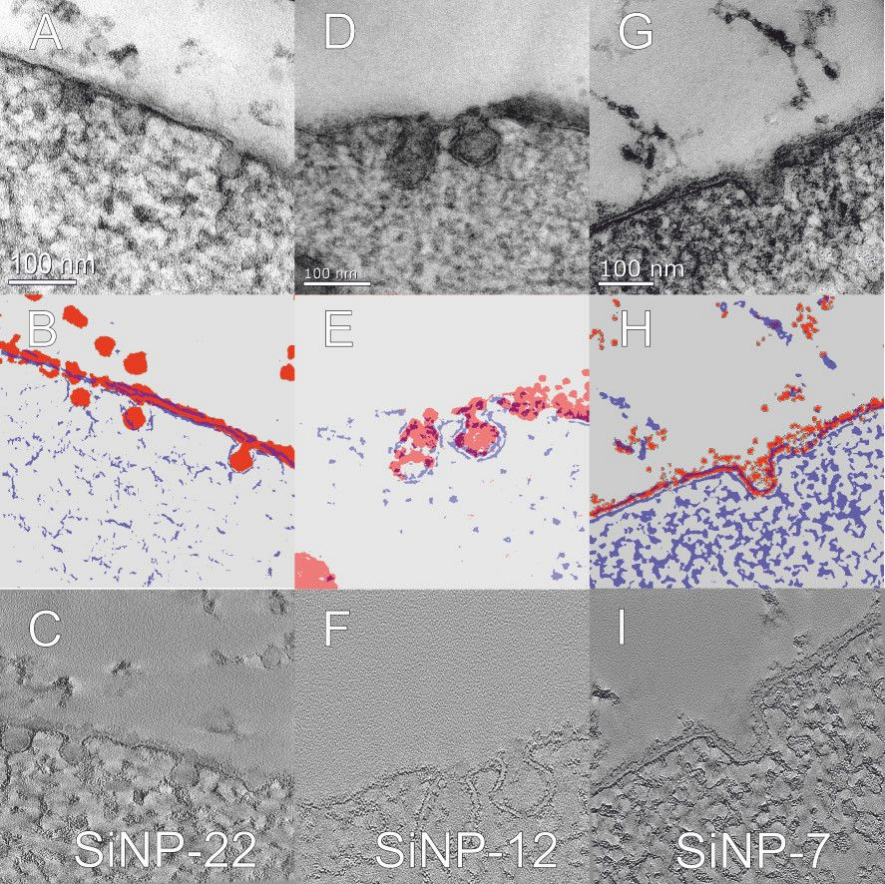
TEM brightfield micrographs of high-pressure frozen hMSC cells exposed to SiNPs at a concentration of *c* = 75 µg·mL^−1^ for 10 min, stained with OsO_4_ and uranyl acetate (micrographs A, D and G show SiNP-22, SiNP-12 and SiNP-7, respectively). Prior and during the incubation, the hMSCs were kept at 4 °C. For clarification purposes the illustrations below (B, E and H) represent the membrane (blue) and the SiNPs (red) as extracted from energy filtered TEM measurements of the above micrographs. Finally, the micrographs in the bottom row show an optical slice from the respective tomography reconstruction (C, F and I).

Furthermore, since all morphological observations yield similar structural information, we checked the uptake morphologies under conditions when all energy-dependent processes in the cell are suppressed [9,11]. For this, we cooled the hMSCs down to 4 °C 10 min prior to incubation and kept the cells exposed to the particles at this temperature for 10 min until high pressure freezing (Figure 11). Again, the uptake morphologies are nearly equivalent to those already observed; individual particle uptake in case of SiNP-22 and concerning SiNP-12 the morphology more or less appears like a transition from the row-like to the tubular structure. For SiNP-7, however, the uptake morphology has changed considerably. The tubular structures found for uptake at 37 °C are gone and only small, spherical cavities can be found along the cell membrane instead. Furthermore, the amount of NPs interacting with the cell membrane is significantly lower in comparison to the same experiment carried out at 37 °C.

## Discussion

In the present study we show that small silica NPs (1) tend to agglomerate in a protein containing environment; (2) reveal a size-dependent mode of uptake into cultured cells, regardless of NP concentration, medium or temperature; (3) show similar uptake morphologies for all investigated cell lines and (4) induce a size-dependent cytotoxicity most likely mediated by a necrotic process. Furthermore, we observed necrotic structures in TEM analysis, e.g., vacuolization and membrane disintegration supporting the evidences of necrotic cell death.

### Agglomeration of NPs in a protein containing environment and their interaction with the cell membrane

All the NPs under examination showed immediate agglomeration when exposed to protein containing medium as determined by DLS (Table 2). However, DLS measurements are dominated by the scattering of the largest objects in the scattering volume. Nonetheless, DLS data yields clear evidence for the coexistence of agglomerates and individual NPs. This can be seen in the TEM micrographs (Figure 2) as well, showing that still some individual NPs exist besides the large agglomerates. Accordingly, a mixture of agglomerates and individual NPs will approach the cell membrane as can be seen from the SEM micrographs displaying the particle-covered cell membrane of a HeLa cell (Figure 3). The agglomeration of silica NPs is driven by the adsorption of proteins to the NP surface [31] followed by a kind of flocculation (Supporting Information File 1, Figure S4 shows the adsorbed proteins on the NP surface). The SEM micrographs reveal that the cell membrane comes into contact with all ”kinds” of NPs; large and small agglomerates and individual NPs. In the examination of ultrastructure we never observed any uptake of large agglomerates. SiNP-7 might be an exception but we cannot differentiate between protein induced agglomeration of NPs and surface adsorption to the cell membrane in this case. However, for the larger NPs the uptake process is dominated by single events and the cluster formation of the NPs prior to their approach to the cell membrane seems to be of minor importance. One might speculate that the protein induced interaction between the NPs is rather weak and hence their interaction with the cell membrane induces a breakage of single NPs from the cluster assemblage.

### Morphology of uptake

Size-dependent uptake of nanoparticles has been well studied in general. It is accepted that different kinds of nanoparticles are taken up by specific cellular endocytic mechanisms depending on the material, surface charge or size of the applied nanoparticle [32–33]. So far, less is known about the modes of uptake mediated by physical properties of nanoparticles in the diameter range below 25 nm. With our morphological observations we clearly see a size dependency of NP endocytosis (Figure 4, Figure 10, Figure 11). The endocytic route for the larger of our examined NPs (SiNP-22 and SiNP-12) can be described as following: First the NPs are taken up as individuals or in small groups and are found in the cytosol with tightly wrapping membrane. Looking at the TEM micrographs of the endosomal vesicles it could be speculated that shortly after incorporation the cell collects several of these NPs in early endosome-like structures and fuses those to a larger endocytic structure (Supporting Information File 1, Figure S8). The observation of intraendosomal NPs with at least some fragments of a membrane surrounding is suggestive of this process (Supporting Information File 1, Figure S8).

The very small silica NPs, however, do not really seem to follow this route. They adhere to the cell membrane and the membrane recess is not rapidly pinched off the cell membrane (Figure 4C and Supporting Information File 1, Figure S7). However, when this structure pinches off the cell membrane, it already forms an endosome-like structure containing a lot of NPs. A final pinch-off of these tubular invaginations is quite likely because we observe endosome-like structures filled with NPs which are detached from the outer cell membrane (Figure 4F and Figure 10E). The fact, that we even found these structures at some distance from the cell membrane rules out the assumption that the detachment is only an artifact due to sectioning a complex three dimensional tubular structure. From a morphological point of view this uptake is similar to the clathrin-independent carrier (CLIC) GPI-protein enriched early endosomal compartment (GEEC) type of endocytic pathway [2]. Furthermore, one can observe that large areas of the cell membrane are covered with NPs (Figure 4C). One can assume that this large area coverage hinders the final vesicle pinch-off from the cell membrane. The additional silica layer on the cell membrane prevents the intimate membrane–membrane contact necessary for the coalescence of the membrane lipid bilayer at the point of pinch-off. Furthermore, the additional silica layer is likely to change the viscoelastic properties of the cell membrane yielding an increased maximum bending radius. Nevertheless, the cell manages to perform the pinch-off which is indicative for an active endocytic process. Moreover, when suppressing any active endocytosis by cooling the cells the tubular morphology disappears and only small cavities were formed instead (Figure 11C). These explanations are yet speculative until the further explicit identification of the proteins that mediate this endocytic process.

On the contrary, the larger silica NPs enter the cell as individuals (SiNP-22; Figure 4A) or groups of some few particles (SiNP-12; Figure 4B). Upon entering the cytoplasm, they were closely wrapped by a membrane. This cooperative uptake of smaller sized nanoparticles has been predicted by Reynwar and Deserno [34] solely based on the physical interaction of NPs with the membrane. It has also been observed lately in an experimental setup for polymersomes and silica NPs [15,34]. Here notably the diameter of the particles was larger but also other parameters differ from our investigation in live cells like, i.e., the Young’s modulus of the polymersomes was much higher compared to the cell membrane. Nonetheless, it is remarkable that quite a little difference in NP size results in completely different uptake morphologies. From molecular dynamics simulations of two curvature imprinting particles on a lipid bilayer membrane we learn, that the membrane mediates either attractive or repulsive forces between the particles [35]. The scaling parameter is the distance versus radius ratio (*d*/*r*) and the imprint depth. Usually, two particles experience repulsive forces which finally would result in the observation of single particle uptake as observed for SiNP-22. Although several NPs might be agglomerated when approaching the cell membrane, the membrane mediated repulsive forces separate them upon wrapping into the membrane. As a result each particle receives an individual membrane wrapping. Only little smaller radii (SiNP-12) results in a smaller imprint depth and hence reduces the repulsive forces between the particles. The separation of the particles cannot be completed but instead the repulsive forces only cause the observed row-like alignment. This explanation is exclusively based on the physical principles of membrane – particle interaction without any active, protein dependent endocytic pathway necessary. This assumption is further corroborated by the observation, that this process still works when all energy-dependent endocytosis mechanisms were switched off by means of cooling (Figure 11A and B).

### Toxicity

Features like cell swelling, blebbing and increased membrane permeability are summed up under the term “oncosis” as a form of cell death in contrast to apoptosis [36]. Its mechanism is thought to be based on a malfunction of the ionic pumps of the plasma membrane that can be evoked by ischemia or toxic agents interfering with ATP generation or increasing the permeability of the plasma membrane. The accumulation of calcium taking the form of insoluble hydroxyapatite (Ca phosphate) inside mitochondria during a necrotic process is a well-known and documented phenomenon during this process [37–38].

A lot of data concerning cytotoxicity of nanoparticles have been documented [17,28,39]. The majority of these studies show that the smaller a particle, the greater the cytotoxicity. This principle was found for SiO_2_ NPs over a large size range. Napierska et al. reported a size-dependent cytotoxicity in the human epithelial cell line EAHY926 showing that 16 nm SiO_2_ NP already induced cell death at lower concentrations in comparison to larger SiNPs (335 nm/104 nm) after 24 h [17]. Further experiments showed that maximum LDH release was already reached after 6 h of exposure at TC_25_ (toxic concentration 25%) concentrations for small particles tested. Similar results were reported by Zhang et al. comparing 80 nm SiNPs with 500 nm SiNPs on HepG2 cells [40]. Those investigated particles affect cell viability and the proliferation potential in a size-ascending-dependent manner. Nevertheless, these data were only focusing on large differences in size and surface-area dependent cytotoxicity and focused on long incubation times of SiNPs in the order of some 24 h.

In our experiments we applied high concentrations of SiNPs and could observe effects on cell viability and membrane integrity already after a few hours. When inspecting the LDH release experiments (Figure 6) one can determine a certain concentration threshold for each particle size at which the HeLa cells show a significant increase of LDH release. All these significant increases happen to be after 2 h of incubation at concentrations of 800, 1600 and 3400 µg·mL^−1^ for SiNP-7, SiNP-12 and SiNP-22, respectively. As already proposed by Bauer et al., surface area may also play a crucial role in cytotoxicity of SiNPs [41]. When scaling the concentration to the particle size one might use the total area of NPs applied to the cells. Assuming spherical particles the total applied area of NPs scales with 1/*r* and the above threshold concentrations yield a total applied surface area of 0.18, 0.26 and 0.32 m^2^·mL^−1^ for the different particles sizes, respectively.

Accordingly, when discussing the cytotoxic effect of high concentrations of NPs we have to consider the consumption of cell membrane, as every single SiNP-22 particle receives a membrane wrapping which is removed from the cell membrane. Our toxic threshold taken from the LDH measurements (Figure 4) corresponds to a surface consumption of 0.32 m^2^·mL^−1^ for SiNP-22. Above this value the membrane is severely damaged and the cell shows leakage like observed in Figure 6. In contrast, the toxic threshold surface area from LDH measurements is 0.18 m^2^·mL^−1^ for SiNP-7, only. However, the surface area interacting with the cell membrane is considerably smaller due to the tubular uptake morphology. This indicates that here the mechanism of cell damage cannot be attributed to membrane consumption and leakage. This is corroborated by the fact that we never observed leaking cells in TEM after incubation with SiNP-7. Consequently, in this case the uptake morphology and the mechanism inducing cell necrosis are different.

The observed fast LDH release is an indicator for a necrosis-based cell death. Moreover, the live cell imaging with the detection of a burst event and the Hoechst staining of the cell nucleus points into the direction of necrosis. Despite of this common conclusion, we analyzed the cleavage of Caspase-3 showing no specific activation of major extrinsic and intrinsic apoptotic pathways at all concentrations and SiO_2_ NP tested (Supporting Information File 1, Figure S11). Here it is to mention that most of selective apoptotic processes are induced only a few hours after exposure of the initiator [42]. It often occurs that apoptotic processes measured after longer time points miss apoptosis as an event as the late apoptotic cells cannot be distinguished from necrotic cells that have not undergone caspase mediated apoptotic cell death leading to false positive results. Altogether, our data indicate, that exposure of silica NPs to an epithelial cervical model system directly leads to necrosis already showing significant effects in small size differences.

Necrosis is also linked to fast mitochondrial damage, which goes along with the depletion of intracellular ATP [43–44]. When the intra-mitochondrial membrane potential is damaged also ATP synthase function is diminished [45]. Furthermore, intracellular calcium levels are increased affecting the calcium signaling of the cell. The observation of calcium agglomerates in the mitochondria (Figure 9) might be the direct consequence of increased cytoplasmatic calcium levels. We found a time- and dose-dependent depletion of ATP showing a significant loss after 2 h of exposure. Interestingly, this was more pronounced for the larger NPs in the lower concentration regime (<1600 µg·mL^−1^). Then the ATP content was partially restored and plateaued between 2 and 4 h of incubation suggesting a down regulated but functional ATP synthase. This effect was also shown by Dong et al. claiming that nanoparticles are potential effectors for the down regulation of ATP without inducing apoptosis but necrosis [46]. In the high concentration regime all examined NPs induced similar ATP depletion levels which finally lead to a complete cellular breakdown after 5 h.

## Conclusion

In our present studies we examined the ultrastructural cell uptake morphologies of rather small particles in a size range between 10 and 25 nm in diameter. Surprisingly, already in this narrow size range clearly differentiated modes of uptake occur. Furthermore, these modes display quite unexpected structural features with single or few NPs tightly enclosed by a membrane (for the larger NPs tested) or resemble the CLIC/GEEC type endocytic pathway (for very small NPs). The latter can be assumed to be a protein mediated active process whereas the former modes can be explained solely by membrane–particle interactions. Amazingly, the uptake morphologies can be reproduced in quite different environmental conditions and cell lines which we would interpret as an indication for a universal uptake process. However, the results we presented in this study need to be extended by further investigations in order to corroborate the discussed assumptions.

These observations may hint at possible reasons for the strong cytotoxic effects occurring upon NP exposure at high concentrations: Membrane disintegration attributed to consumption of the cell membrane. However, this proposed mechanism needs stronger corroboration by further investigation.

Generally, the cytotoxicity of NPs is believed to be inversely proportional to the particle diameter. However, we struggle to clearly rank our NPs regarding their cytotoxic potential based on our experimental observations. The LDH assay (Figure 6) ranks SiNP-12 and SiNP-7 to be most toxic whereas life cell imaging (Supporting Information File 1, Figure S10) points at SiNP-12. From the ATP measurements (Figure 7) no clear ranking statement is possible. This confusion in correlation between NP size and cytotoxicity may be attributed to the fact that for the smallest particles the uptake mode flips from passive intrusion to protein mediated endocytosis.

## Materials and Methods

### Amorphous silica nanoparticles and particle characterization

The study was performed by using uncoated, amorphous silica nanoparticles in aqueous dispersion. LUDOX SM-30 (ca. 7 nm diameter; 20 wt %; obtained from Grace Davison, U.S.A.), LUDOX HS-30 (ca. 12 nm diameter; 30 wt %) and LUDOX TMA (ca. 22 nm diameter; 34 wt %) (both obtained from Sigma-Aldrich) were characterized by TEM and DLS prior to application. Solid content of the pristine dispersions was determined gravimetrically. NP dispersions for cell applications were diluted with milli-Q water to achieve the desired concentration.

Dynamic light scattering (DLS) experiments were performed on an ALV instrument consisting of a goniometer and an ALV-5000 multiple-tau full-digital correlator with 320 channels. A helium–neon laser JDS Uniphase with a single mode intensity of 25 mW operating at a laser wavelength of λ_0_ = 632.8 nm was used as light source. Details of the measurements and data evaluation procedure are described in the literature [47]. For light scattering experiments, sample dispersions at concentrations of *c* = 5 × 10^−2^ wt % were prepared by dilution of the previously described stock dispersions. All measurements were carried out at a temperature of *T* = 20 °C.

For TEM measurements the samples were prepared by diluting the as supplied sample dispersion with water yielding a solid content of approximately 0.1% and subsequently applied to a carbon coated TEM grid. Excess solution was blotted off and the sample was dried at ambient conditions. For automated analysis the TEM micrographs were processed using Gaussian blur filtering with 2 pixels radius, background subtraction, threshold setting and a final watershed algorithm. Particle detection was restricted between an upper and lower cut off in particle radius and to a circularity greater than 0.7. This way only separated particles were considered for measurement.

### Cell culture

For TEM experiments, all different cell lines were seeded at a concentration of 30,000 cells·cm^−2^ and cultivated for 24 h before nanoparticles were added. For biochemical assays, an initial cell density of 15,000 cells·cm^−2^ was applied and left to attach for 24 h. In order to use high pressure freezing (HPF) as the fixation technique for TEM, cells were seeded on 3 mm plasma-sterilized sapphire discs (M. Wohlwend GmbH, Sennwald, Switzerland) that had been covered with a 20 nm carbon layer before usage. All cell types were kept in a humidified incubator with 5% CO_2_ at 37 °C (Labotec, Göttingen, Germany). The culture medium of HeLa cells consisted of Dulbecco’s Modified Eagle’s Medium (DMEM) (Life Technologies, U.S.A.) supplemented with 10% fetal calf serum (FCS) (Invitrogen, Karlsruhe, Germany), 100 units penicillin and 100 µg·mL^−1^ medium streptomycin (Life Technologies) and 1 mM pyruvate (Life Technologies). Osteosarcoma (U2OS) cells were grown in DMEM with an addition of 10% FCS, 1% penicillin/streptomycin and 1% MEM NEAA. For B16-F10 cells RPMI 1640 medium (Life Technologies) was used containing 10% FCS, 1% 1M HEPES, 1% MEM NEAA, 1% pyruvate and 1% penicillin/streptomycin as supplements. Caco-2 cells were kept in EMEM (Lonza) supplemented with 10% FCS, 1% Glutamax (Life Technologies) and 1% penicillin/streptomycin. Primary hMSCs were cultivated using MEM Alpha (Lonza) with an addition of 20% FCS, 1% penicillin/streptomycin, 1% pyruvate and 0.6% ciprofloxacin. On the second day of cultivation of each cell type, a calculated amount of SiNP suspension was added to the cell culture medium, yielding the final concentration for exposure. For transmission electron microscopy and for cytotoxicity assays, cells were incubated with SiNPs for 10 min to 24 h at 37 °C and 5% CO_2_ before further processing.

To check if the observed particle engulfment is an energy-dependent process or not, cells were cooled down to 4 °C for a 10 min period prior to incubation with nanoparticles. The incubation was done at the same temperature for 10 min before sample preparation for TEM via HPF. We did not choose longer cooling times as the change of cell morphology on the ultrastructural level is already marked after this time with cells showing many and huge vacuoles that were not present before.

### Scanning electron microscopy (SEM)

SEM analysis was performed using a Hitachi SU8000 scanning electron microscope operated at an acceleration voltage of 1 kV. Sample preparation for SEM examination was performed using critical point drying (CPD) of the cultured cells.

### Sample preparation for TEM analysis

For TEM analysis, the cells were loaded with different concentrations of NPs ranging from 75 to 3400 µg·mL^−1^. After incubation for the respective time, the cells were cryo-fixed within a few milliseconds at a pressure of 2000 bar under liquid nitrogen using a high-pressure freezer Compact 1 (Wohlwend GmbH, Switzerland). Freeze-substitution was conducted using a Leica EM AFS 2 device (Leica Microsystems, Germany). Here, the substitution/staining medium (acetone p.a., 0.2% osmium tetroxide, 0.1% uranylacetate and 5% water) was pre-cooled to −90 °C before samples were added. Finally, the samples were embedded in EPON 812 and sectioned at room temperature using a diamond knife. For elemental analysis, some of these specimens were additionally coated with a thin carbon layer in order to increase their stability against beam damage.

### Transmission electron microscopy (TEM)

Examination of the thin sections was conducted with a FEI Tecnai F20 transmission electron microscope (FEI, USA) operated at an acceleration voltage of 200 kV. This microscope was equipped with a Gatan tridiem image filter (Gatan Inc., USA) and an energy dispersive X-ray fluorescence (EDX) detector (EDAX Inc., USA) for analytical element measurements. Conventional bright field images were acquired using a Gatan US1000 slow scan CCD camera (Gatan Inc., USA).

### Inelastic dark field imaging for NP identification

Since the contrast of the silica particles in bright field imaging was too low for unambiguous identification, we applied inelastic dark field imaging techniques for the visualization of silica nanoparticles. Inelastic dark field imaging was conducted using the image filter/electron energy loss spectrometer. For identification of silicon rich areas we selected an energy loss of 80 eV with the energy selecting slit width set to 20 eV. With these settings the silicon rich areas in the inspected thin sections appeared significantly brighter compared to the environment. Choosing an electron energy loss window at 80 ± 10 eV for imaging the silica particles was necessary because of the presence of uranium stain. The uranium O_45_ absorption edge coincides with the silicon L_23_ absorption edge which are both located around 95 eV electron energy loss (Supporting Information File 1, Figure S1). Accordingly, the inelastic dark field images (shown in Supporting Information File 1, Figure S8) reflect in principle a local superposition of the electron density and the plasmon interaction cross section of the specimen. As a result, the silica nanoparticles will appear brighter compared to the surrounding tissue. However, one has to keep in mind that the embedded section has a thickness in the order of approximately up to 100 nm and an individual silica particle can be as small as 7 nm. This limits the detectability of small, separated silica NPs in the cell interior. For further image processing, the inelastic darkfield micrographs were segmented to differentiate Si containing pixels from background applying a semi-automatic image processing procedure. For image segmentation we used the trainable segmentation package (Image J) [48–49] and finally prepared a color coded overlay of the silicon rich areas to the TEM brightfield image. Membrane segmentation was done in a similar way, using principal components analysis of spectrum image series [50] prior to segmentation.

### EELS/EDX

For spatially resolved elemental analysis we applied EDX spectroscopy and electron energy loss spectroscopy (EELS). For EDX measurements the TEM was operated in scanning mode (STEM) using a high angular, annular dark field detector (HAADF). Areas of interest were than exclusively excited by the electron beam while measuring the EDX spectrum yielding the chemical composition of the respective area. EELS measurements were done in conventional TEM mode. In order to extract the required local information, the electron beam of the microscope was focused on the area of interest solely by adjusting the C2 condenser lens. Acquisition of the EELS spectrum was then done in diffraction mode using a 1 mm spectrometer entrance aperture.

### LDH release assay

Cell membrane damage was quantitatively determined using an LDH release assay according to manufacturer’s instructions (Abcam LDH-Cytotoxicity Assay Kit II, (Abcam, United Kingdom)). Briefly, HeLa cells with a density of 15,000 cells·cm^−2^ were seeded on a sterile 96-well-plate (Corning, New York City, U.S.A.) in triplicates and incubated for 20 h. After particle load and incubation, cells were centrifuged for 10 min at 600*g*. Then, 10 µL of cell culture supernatant was transferred into a fresh 96-well plate, mixed with 100 µL of the LDH reaction mix and incubated for 60 min at room temperature. Further on, the absorbance of the samples was measured at 450 nm in a plate reader and reference values were measured at 650 nm (Tecan Infinite M1000 PRO (Tecan, Switzerland)). The mean value of triplicates was calculated, normalized to the LDH positive control and is depicted as the quotient of the following formula (Abs: Absorbance):


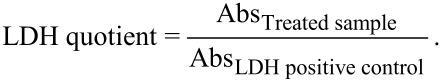


### Measurement of intracellular ATP content

Intracellular ATP content was measured according to the kit instructions of CellTiter-Glo^®^ Luminescent Cell Viability Assay (Promega, U.S.A.). Briefly, 15,000 HeLa cells·cm^−2^ were seeded on a 96-well plate. After particle incubation, cell lysis was performed by the addition of CellTiter-Glo^®^ reagent for 2 min. An additional incubation time of 10 min allows the reaction to enhance the chemiluminescent signal. The luciferase-catalyzed reaction is stated as followed: ATP + Luciferin + O_2_ → AMP + PP_i_ + Oxyluciferin + CO_2_ + Light. Light emission of samples was recorded in a luminometer (Tecan Infinite M1000 PRO) at an integration time of one second. The signal mean value of triplicates was calculated of each sample and is depicted as the quotient of RLI (mean, sample)/ RLI (mean, untreated negative control) (RLI: Relative luminescent intensity).

### Statistics

Data are presented as means ± SD of triplicates. Data were normally distributed (F-test). For comparison of the LDH and ATP data unpaired t-tests were used. The statistical analysis was conducted with the software Graph Pad Prism 5.03 (Graph Pad Prism Software, U.S.A.).

## Supporting Information

File 1 Additional experimental data.
