# Supplementary material for: On the pathway of cellular uptake: new insight into the interaction between the cell membrane and very small nanoparticles
Source: Beilstein J Nanotechnol. 2016 Sep 16;7:1296–311. doi: 10.3762/bjnano.7.121 (PMC5082453; doi:10.3762/bjnano.7.121)
Supplement: File 1 — Additional experimental data. [file Beilstein_J_Nanotechnol-07-1296-s001.pdf]

Supporting Information

for

On the pathway of cellular uptake: new insight into the interaction between the cell membrane and very small nanoparticles

Claudia Messerschmidt[‡,1], Daniel Hofmann[‡,1], Anja Kroeger[1,2], Katharina Landfester[1], Volker Mailänder[1,3] and Ingo Lieberwirth[*1]

Address: [1]Max Planck Institute for Polymer Research, Ackermannweg 10, 55128 Mainz, Germany, [2]Ostwestfalen-Lippe University of Applied Sciences, Liebigstr. 87, 32657 Lemgo, Germany and [3]Dept. of Medicine III, Hematology, Oncology and Pneumology, University Medical Center of the Johannes Gutenberg University Mainz, Langenbeckstr. 1, 55101 Mainz, Germany

Email: Ingo Lieberwirth - lieberw@mpip-mainz.mpg.de

* Corresponding author:

‡ Equal contributors

Additional experimental data

Conversion between mass- and particle number concentration

For the specification of the incubation concentrations it is reasonable to be as precise as possible. Determination of the solid content of the incubated solution is easy and yields the most defined value in units of, e.g., mass per volume of dispersion or in weight percent. However, since the number of particles or their total surface area is of special interest with regard to toxicity, one has to consider the particle size and especially take the particle size distribution into account. This constitutes the primary problem for the conversion from weight units to particle numbers. DLS and TEM give slightly different results for the average particle radii of the applied NPs SiNP-22, SiNP-12 and SiNP-7. Accordingly, for a particle number based specification of incubation concentration one has to decide for either the TEM or the DLS average particle radius. In Table S1 we give the conversion between mass concentration and the respective particle concentration depending on the respective mean particle radius r_n from TEM and mean hydrodynamic radius R_H from DLS measurements (Table S1) at the basis of a bulk density of silica of 2.65 g/cm^3.

Table S1: Conversion between mass-concentration and particle concentration as calculated from the average particle radius measured by TEM and DLS based on a silica density of 2.65 g/cm^3.

	mass concentration [µg/mL]	particle concentration (TEM) [1*10^{10}/mL]	particle concentration (DLS) [1*10^{10}/mL]
SiNP-22	1	5.2	1.3
SiNP-12	1	26.3	9.0
SiNP-7	1	72.1	6.8

Inelastic darkfield imaging and identification of silica particles within the thin sectioned specimens:

Identification of the silica NPs worked best for imaging in inelastic darkfield with ΔE of 80 eV and a slit width of 10 eV, whereas silicon mapping according to the three window method [1] failed. This is attributed to the fact, that the specimen contains uranium from the staining protocol. Silicon and uranium both have an adsorption edge near 100 eV energy loss and hence an unambiguous mapping of silicon is impossible for our kind of samples. Nonetheless, we found that in the pre-edge energy loss area the background decay curve differs for the silica rich and the uranium containing surrounding (Figure S1) which enables the "pre-edge" darkfield identification imaging.

The spectra in Figure S1 were taken from the same sample area with the electron beam focused by adjusting the C2 lens and the beam shift to an area containing mainly silica particles (green) or only cell tissue without any silica (red).

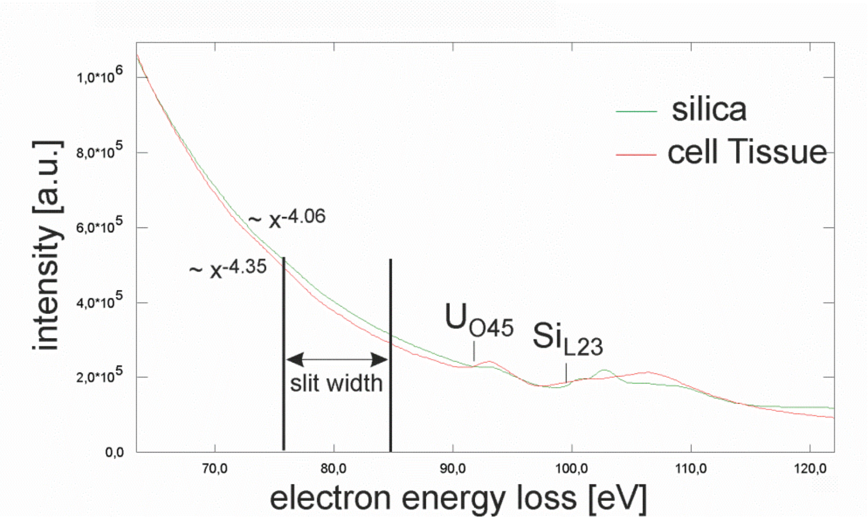

Figure S1: Electron energy loss spectra (EELS) of the cell tissue (red) and silica particles (green).

Identification of 7 nm sized silica NPs

Figure 7G–I and Figure S9 gives further examples of the interaction of the smallest NPs with the cell membrane in addition to Figure 4E,F. For the localization of these NPs one has to keep in mind that the thickness of the microtome sections is with approximately 80 to 100 nm considerably larger than the NP diameter. Assuming an isolated silica particle within the thin section, the silica signal in the

EEL spectrum contributes only with a factor of 0.1 to the entire spectrum at the NP location. This makes it rather difficult to unambiguously identify those isolated silica NPs within the cell interior. Accordingly, we can´t exclude that, besides those already identified, further individual small silica NPs reside within the cell interior.

STEM of uptake morphologies

Figure S2 shows a compilation of STEM micrographs of HeLa cells with high loadings of SiNP-22. The NPs particles permeate into the cell and are tightly covered with a membrane afterwards. In Figure S2 E and F one can observe a NP just at the last state of the permeation process: The uptake of the NP is nearly completed, the NP is covered with a membrane but still sticks to inner cell membrane. NPs outside the cell do not show any indication of an additional surrounding membrane, as can be seen in Figure S2C and D. However, those particles exhibit a granular surface with approximately 1 nm large bright spots. It is very likely, that this is due to the protein adsoption on the NPs. Figure S4 shows some of those external NPs exemplarily for SiNP-22 and Si-NP12. In Figure S4A one can see the lipid bilayer of the cell membrane as well. The NP below the membrane is already taken up by the cell whereas the NPs in the upper part of the micrograph are still outside with the protein coverage visible as tiny bright spots on the NP. Finally, Figure S3 shows some examples of the row-like uptake morphology of sample Si-NP12.

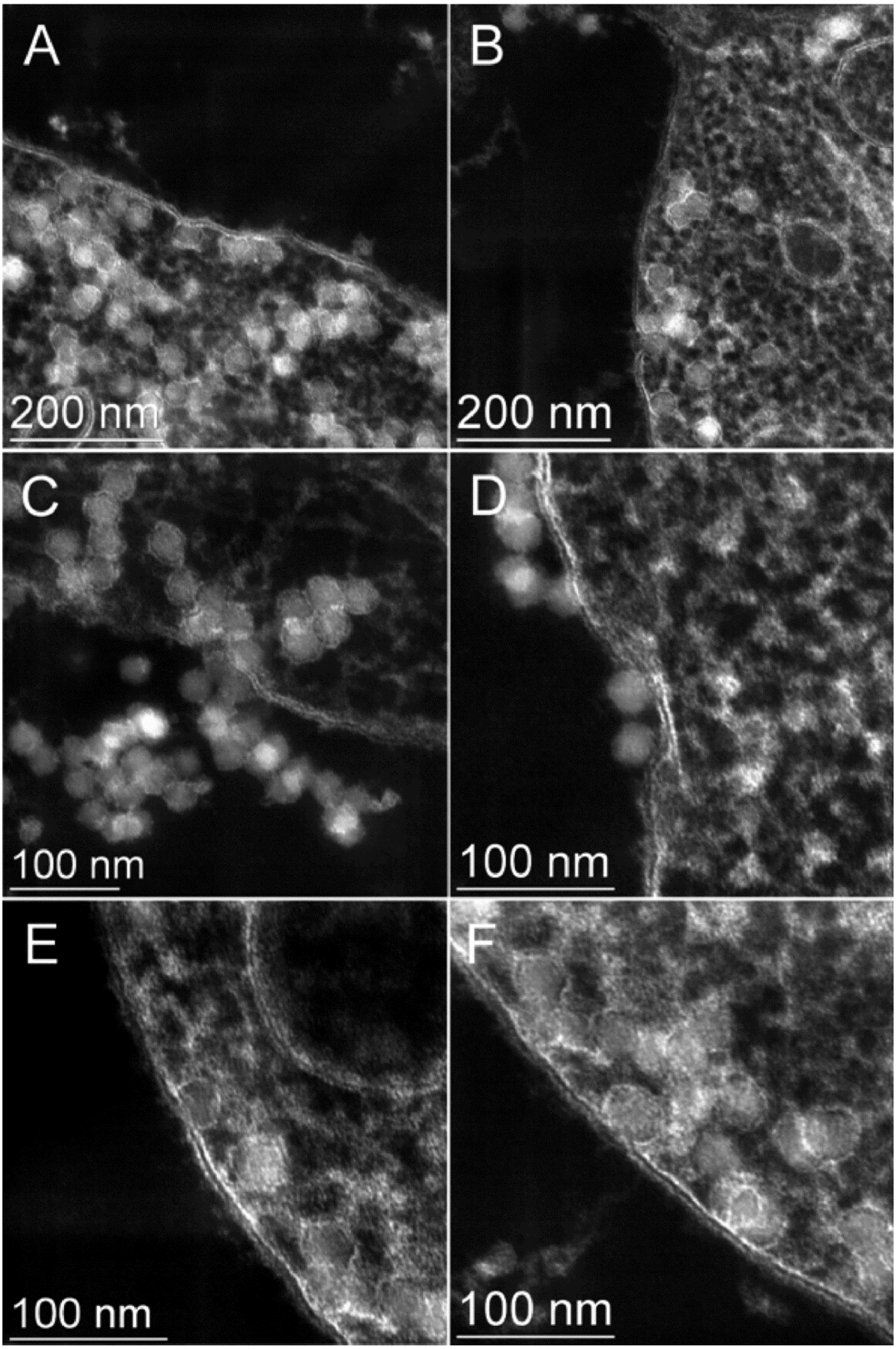

Figure S2: STEM micrographs illustrating the uptake process of SiNP-22 particles close to the cell membrane. Each NP, that enters the cell, is tightly covered by a membrane. The micrographs show HeLa cells incubated with 200 µg/mL SiNP-22 for 2 h before high presure freezing.

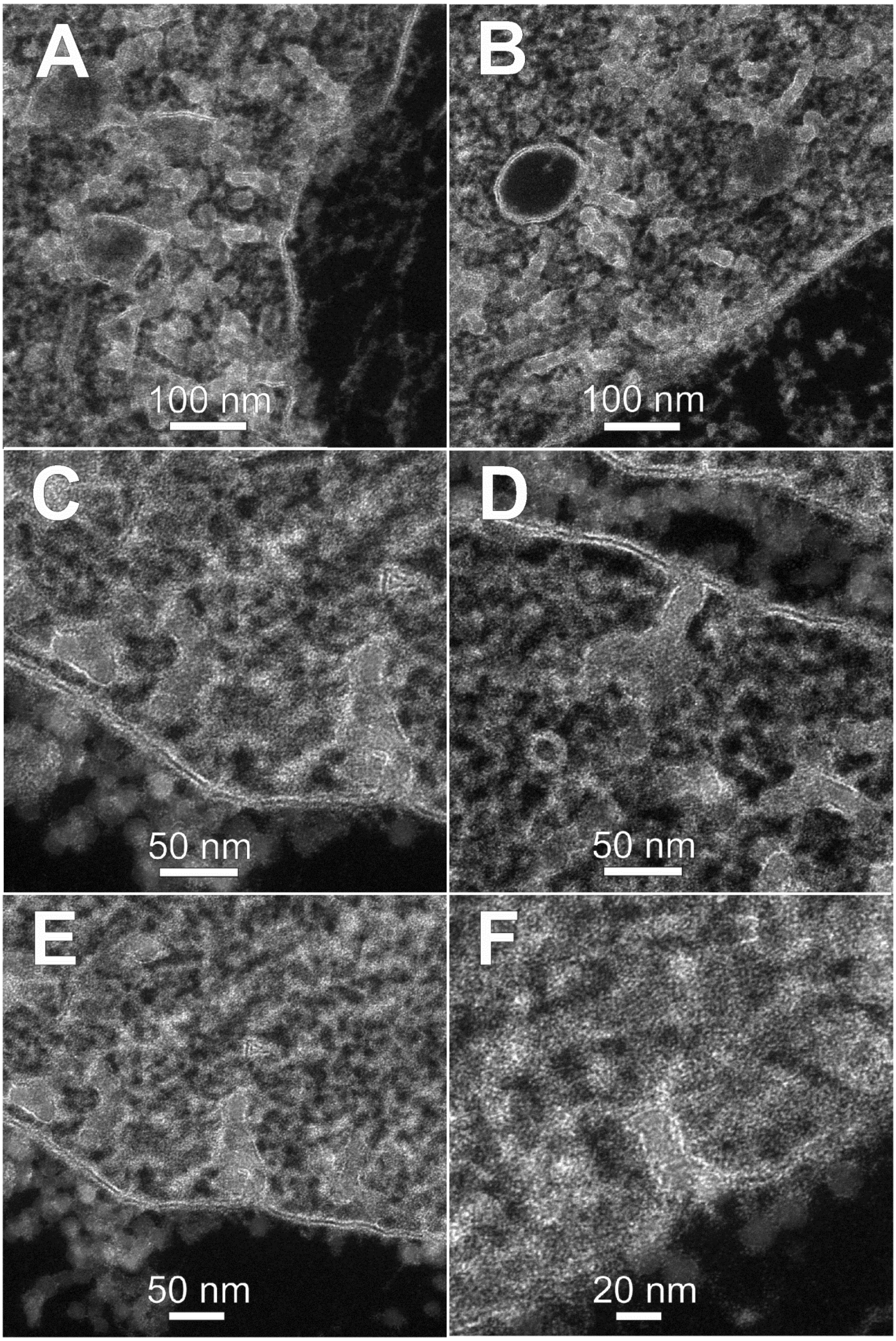

Figure S3: STEM micrographs illustrating the uptake process of SiNP-12 particles close to the cell membrane. The NP´s enter the cell in small groups in a row-like arrangement. Experimental conditions same as in Figure S2

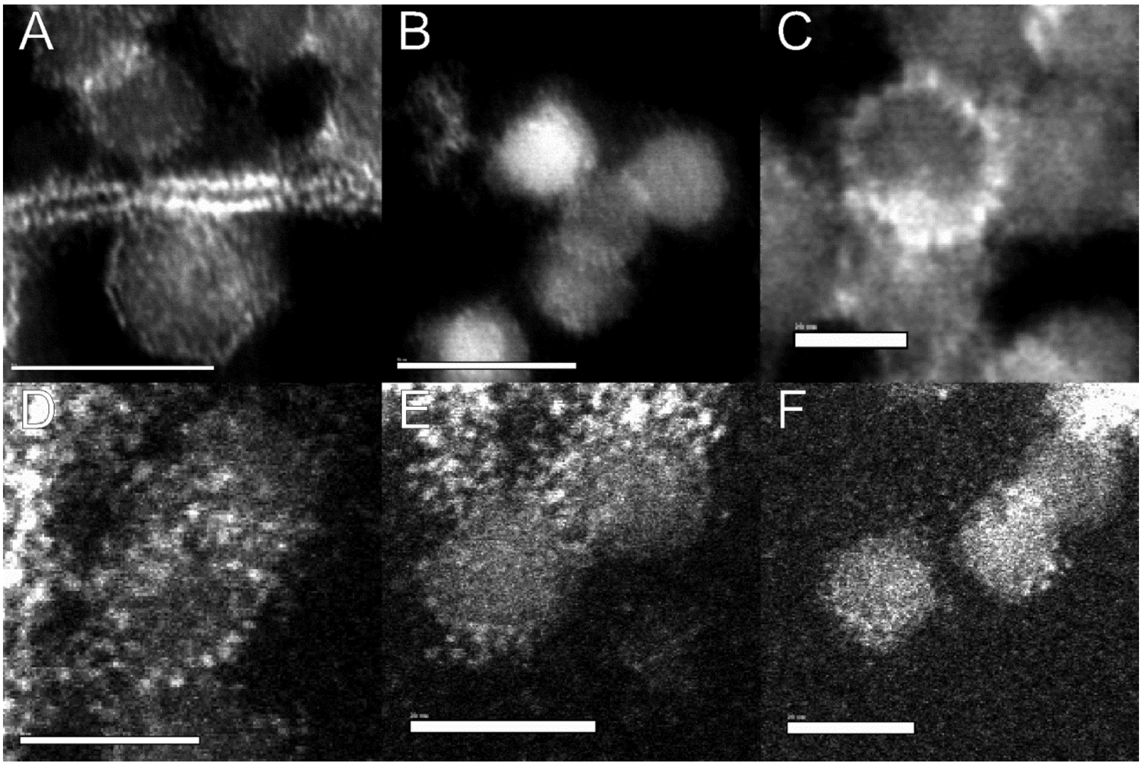

Figure S4: STEM micrographs showing SiNP-22 (A – C) and SiNP-12 (D – F) outside the cell membrane. The distinct bright spots on the silica particles can be interpreted as proteins that adsorbed onto the particle surface. The contrast has been optimized for illustration. (Scale bars: 50 nm (A,B) and 20 nm (C – F)).

Uptake morphologies observed for different cell lines

In addition to the HeLa cells, we checked for the uptake morphologies of another 4 different cell lines: hMSC, U2OS, Caco-2 and B16-F10. Particle incubation time was 30 min at a NP concentration of 2000 μg·mL^{-1}. The TEM observations are similar to those observed for HeLa cells: single particle uptake for Si-NP22 (Figure S5), rowlike structures for Si-NP12 (Figure S6) and tubular membrane morphologies for Si-NP7 (Figure S7). For all the cell lines we could easily find NPs in the cytosol except for Caco2 cells treated with Si-NP22 (Figure S5 D). This might be attributed to the short incubation time of 30 min.

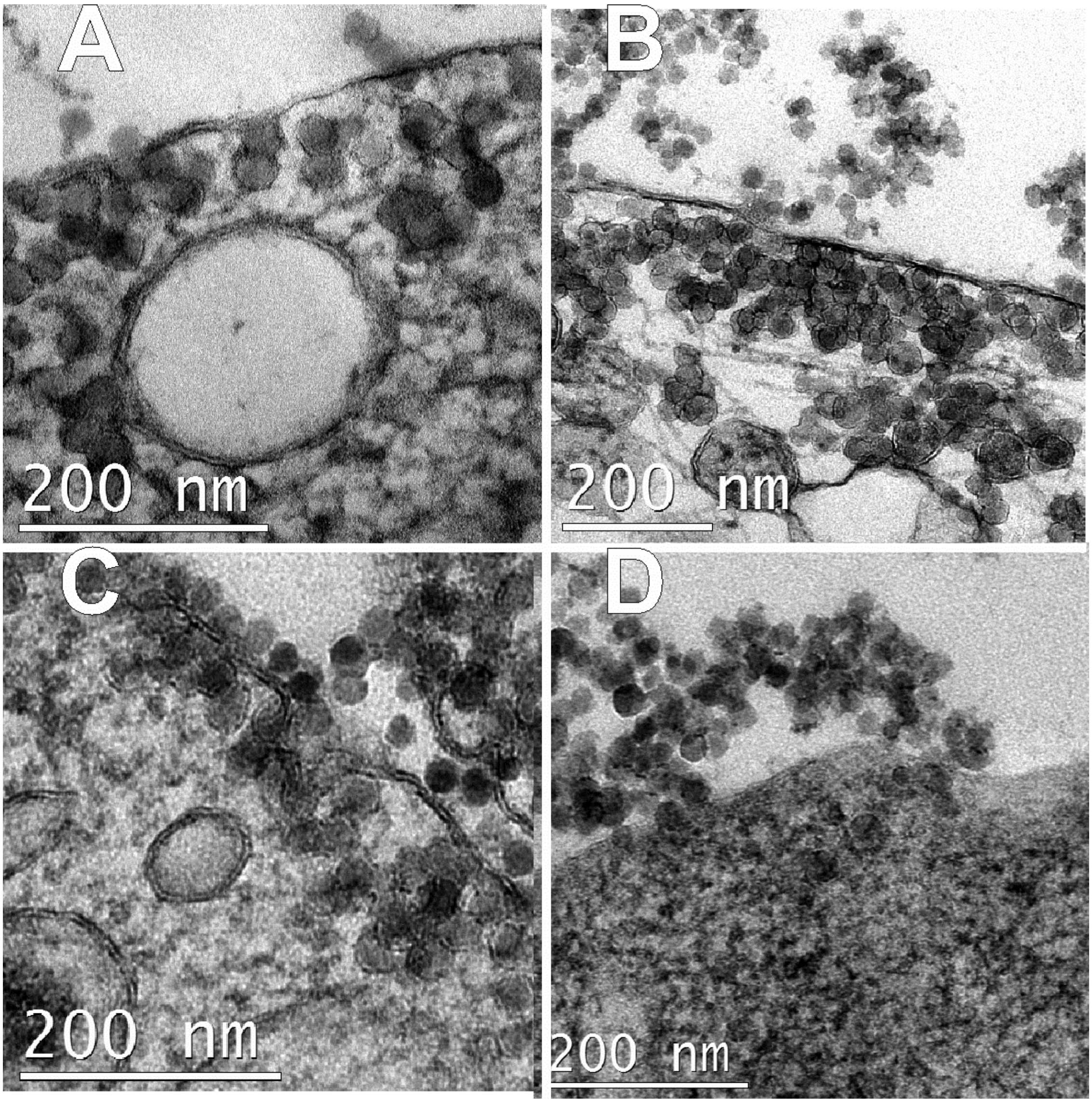

Figure S5: TEM brightfield micrographs showing the membrane morphologies of uptake of SiNP-22 for hMSC- (A), U2OS- (B), B16-F10- (C) and Caco-2 cells (D). The incubation time before high pressure freezing was 30 min and the particle concentration was 2000 µg/mL.

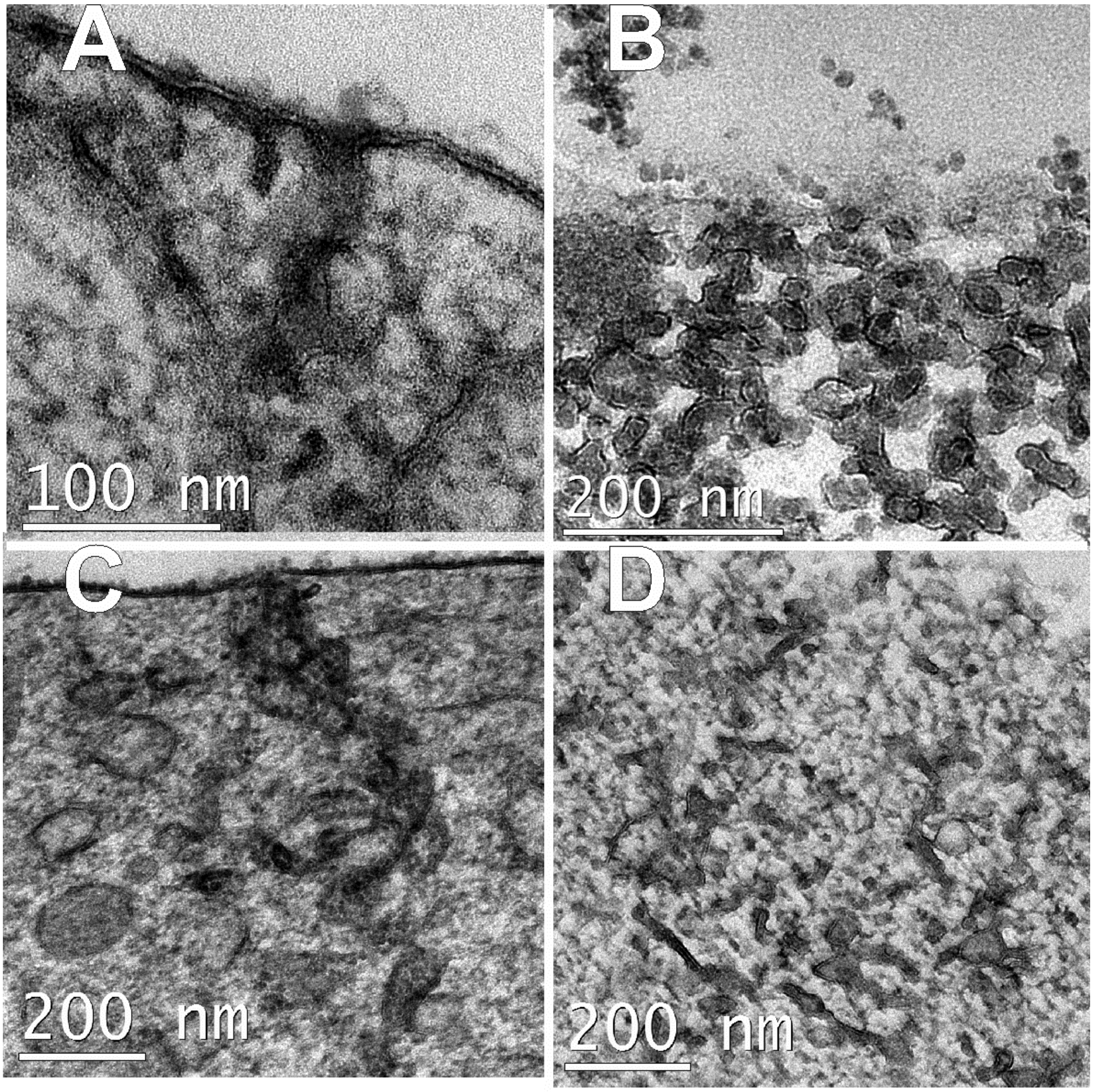

Figure S6: TEM brightfield micrographs showing the membrane morphologies of uptake of SiNP-12 for hMSC- (A), U2OS- (B), B16-F10- (C) and Caco-2 cells (D). The incubation time before high pressure freezing was 30 min and the particle concentration was 2000 µg/mL.

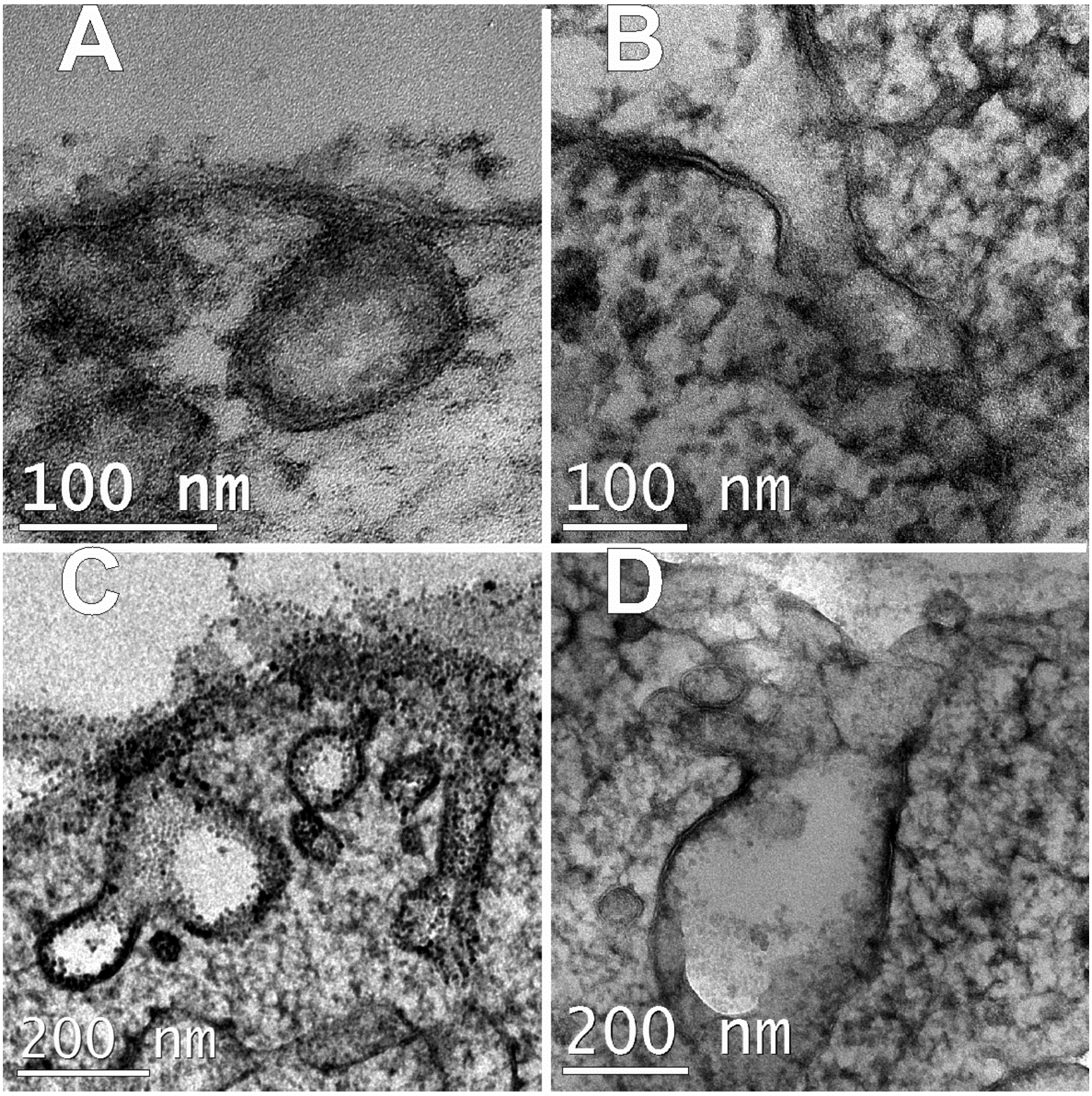

Figure S7: TEM brightfield micrographs showing the membrane morphologies of uptake of SiNP-7 for hMSC- (A), U2OS- (B), B16-F10- (C) and Caco-2 cells (D). The incubation time before high pressure freezing was 30 min and the particle concentration was 2000 µg/mL.

NP uptake morphologies at low incubation concentrations

We started our TEM study using the human epithelial carcinoma cell line HeLa and incubated the cells with 200 $\mu g \cdot mL^{-1}$ of SiNP-22, SiNP-12 and SiNP-7 for two hours in DMEM with serum FCS.

As similarly reported by others [2], we found Si-NP22 and Si-NP12 in vesicles resembling those along the endo-lysosomal pathway (see Figure 3A–F). But in addition to this expected

behavior, we observed that some of the intravesicular 12 nm silica particles showed a tight, individual membrane. Figure S8 shows a closer comparison of the brightfield and inelastic darkfield TEM micrographs, representing the local electron density and silicon concentration, respectively. Whereas for NP 1 the measured diameters for the electron density and the silicon concentration differ by 5 nm, these diameters are nearly similar for NP 2. This has to be interpreted as that NP 1 has an additional membrane whereas NP 2 does not.

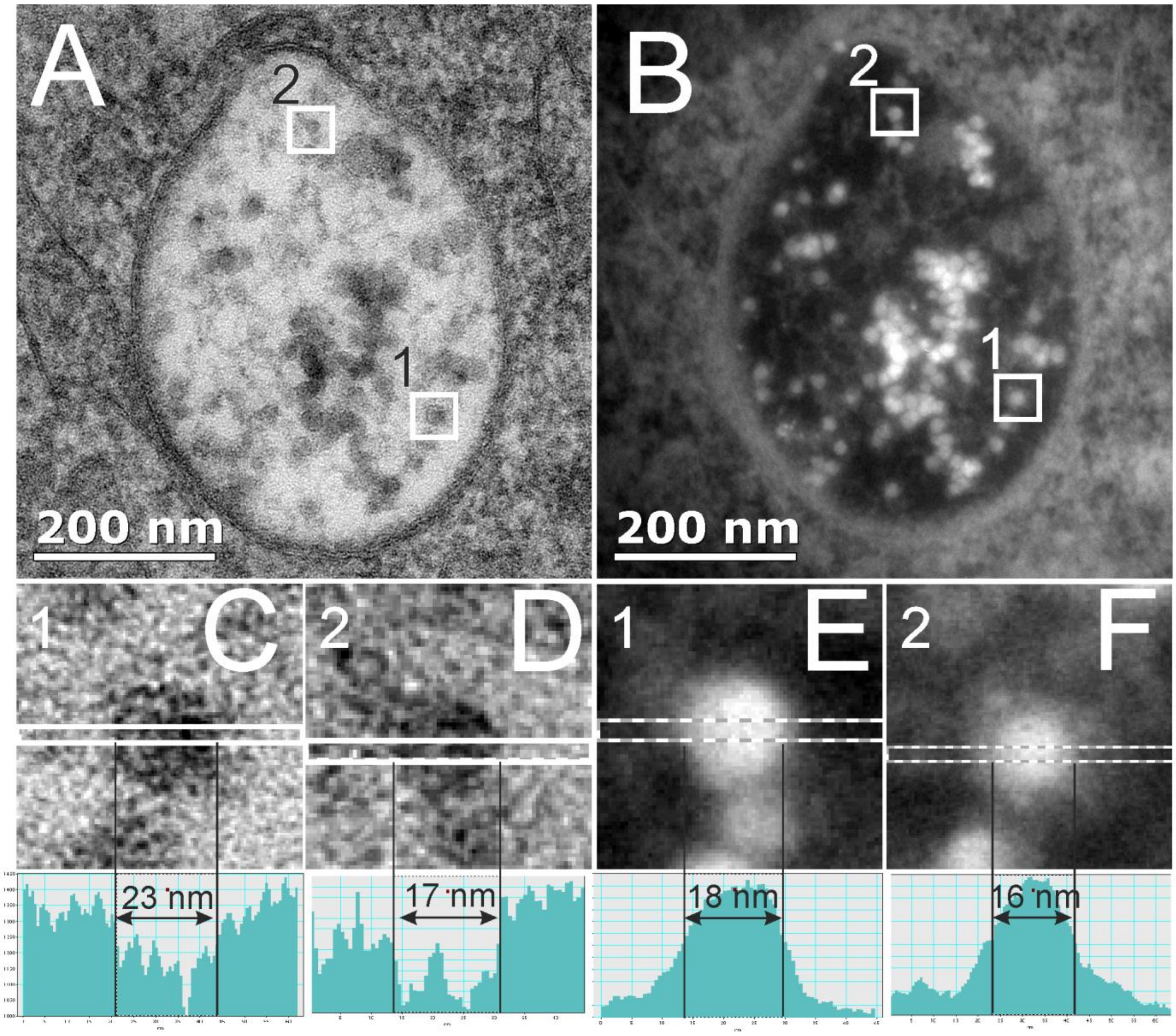

Figure S8: (A,B) TEM micrographs of high-pressure frozen HeLa cells treated with 200 µg·mL^{-1} of SiNP-12 for 2 h (A: bright field, B: inelastic dark field, ΔE = 80 eV); (C,D,E,F) close-ups of the particles marked in A and B with analysis of the particle diameters in the bright field versus the inelastic dark field. The diameter was taken using the full-width half maxima of the intensity profiles. In the TEM brightfield the particle marked in area 1 gives the impression of having an additional membrane or bio-corona whereas the particle

marked in area 2 does not. This can be corroborated by taking the intensity profile of either the brightfield or the inelastic darkfield micrograph. Whereas the brightfield micrograph profile yields a value proportional to the local electron density of the material, the inelastic darkfield is expected to reflect the local silicon concentration. When comparing both profiles (brightfield and inelastic darkfield) one can see that for NP 2 these two profiles / diameters coincide whereas for NP 1 the darkfield diameter appears to be 5 nm larger than the actual diameter of the silicon particle, indicative of an additional membrane around this silicon particle.

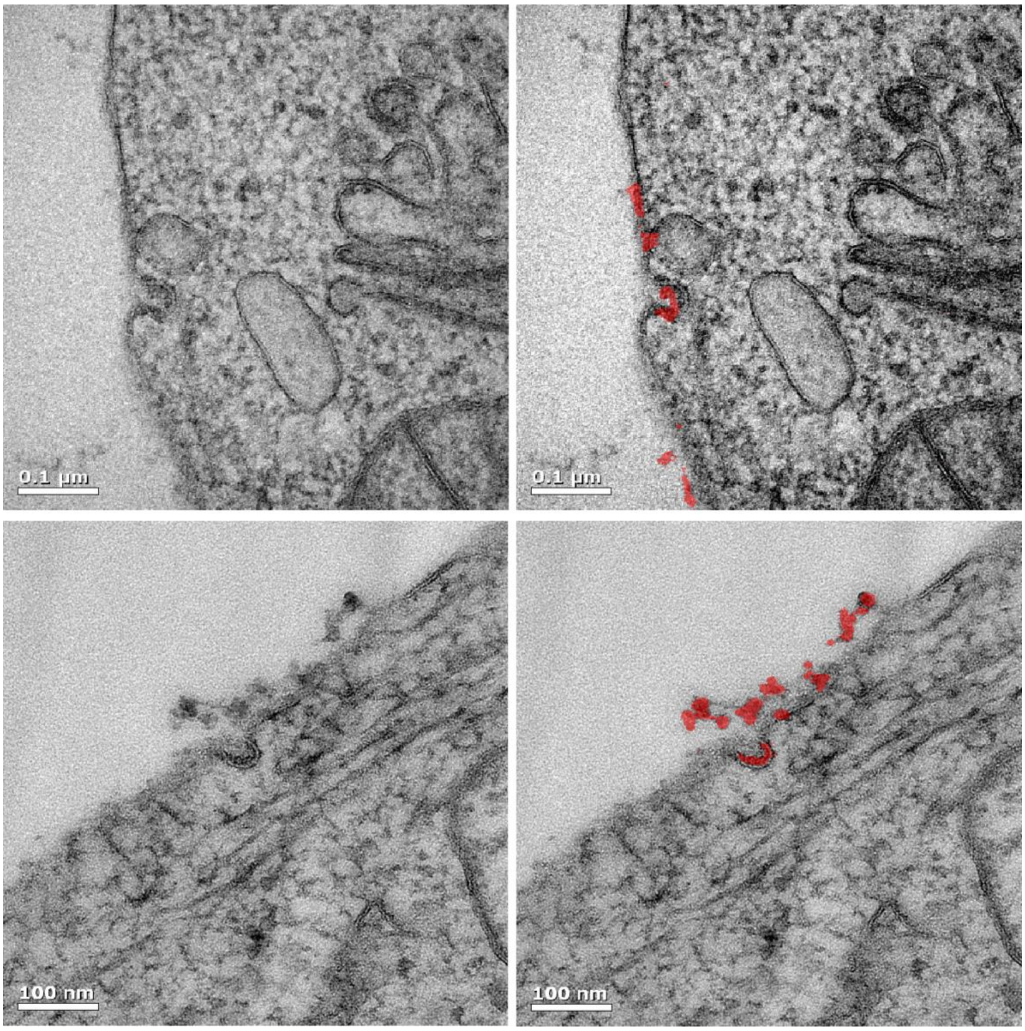

Figure S9: TEM brightfield micrographs of HeLa cells incubated with SiNP-7 at 200 µg/mL for 2 h. The micrographs on the right side display an overlay of the conventional brightfield with the results from the energy filtering to identify silicon rich areas (in red).

Confocal laser scanning microscopy / life cell imaging of U2OS cells

For life cell imaging experiments we used U2OS cells stably expressing TagRFP-labeled tubulin (BioCat, Heidelberg, Germany). TagRFP is a fluorescent protein with adsorption and emission maxima are at 555 and 584 nm, respectively [3]. After addition of different high concentrations of NPs the morphology of cells was observed for 2 h by confocal laser scanning microscopy (cLSM). As can be seen in the micrographs (Figure S10), cell morphology changes to a spherical shape after a certain time depending on NP type, concentration and size. This spherical shape indicates that cells are about to detach from the substrate, a reaction commonly observed when environmental parameters become less suitable for cell growth [2]. The effect of NP treatment is significantly different for the three applied sizes of NPs. For SiNP-12 the cellular reaction is most obvious whereas a change in morphology is not induced by the addition of SiNP-7.

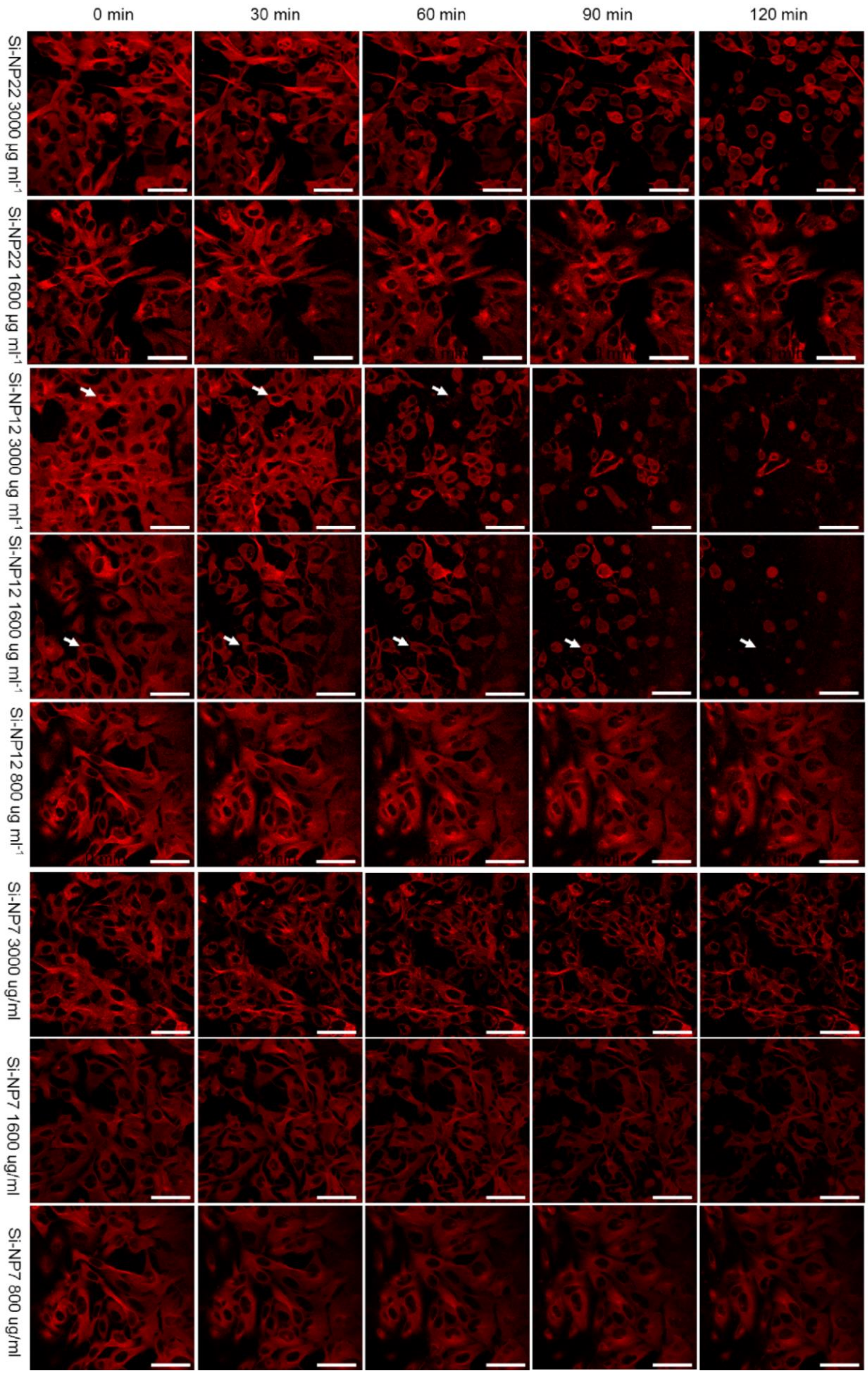

Figure S10: cLSM micrographs of U2OS cells stably expressing TagRFP-labeled tubulin. The cells were incubated with different types and concentrations of SiNPs as indicated on the left hand side. Each experiment was observed for 2 h with image acquisition every 30 min. White arrows indicate bursted cells as an example.

Caspase-3 Western Blotting

The investigation of Caspase-3 cleavage as an effect of exposure to SiNPs was tested by western blotting. Briefly, 15,000 Hela cells cm^{-2} were seeded on a 6-well plate (Gibco, U.S.A.). After 4 h of exposure with different concentrations, whole cell lysates were harvested from HeLa cells by using the NuPAGE® LDS-sample loading buffer (Life Technologies, U.S.A.). Afterwards, equal amounts of the particle-exposed lysate and the staurosporine-treated positive control (incubation for 4 h; 1 µM; (Sigma-Aldrich)) were loaded onto a 10% NuPAGE gel. Soluble protein fractions were run and dry-blotted to a poly(vinylidene fluoride) (PVDF) membrane for 5 min at 23 V. The PVDF membrane was then processed for immunostaining with components of the Novex® Western Breeze™ Immunodetection Kit (Life Technologies). Membranes were stained with Caspase-3 antibodies (Abcam, U.S.A.) and were developed in a LAS-3000 imager (Fujifilm, Japan).

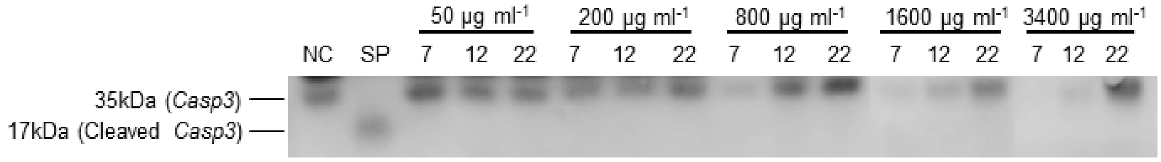

Figure S11: Analysis of activation of Caspase-3 by Western Blotting. Hela cells were treated with the indicated SiO_2 NP and concentrations for 4 h and then analyzed by Caspase-3 specific western blotting. Equal amounts of protein were loaded onto the gel. Samples with high nanoparticle exposure do not show any uncleaved Caspase-3 probably due to progressed protein degradation (NC: Untreated negative control; SP: Staurosporine treated positive control).

Hoechst 33258 staining

To investigate chromatin condensation as an effect of apoptosis, we seeded 15 000 HeLa cells per cm^2 on 6 well plates and exposed them to our nanoparticles. As a positive control for chromatin condensation, cells were incubated with 2 µM staurosporin (Sigma-Aldrich, U.S.A.) for 6 h to induce Caspase-3 and the endonuclease system. Post incubation, cells were stained with 10 $\mu g \cdot mL^{-1}$ Hoechst 33258 for 15 min at RT. Cells were fixed and forwarded to microscopic analysis.

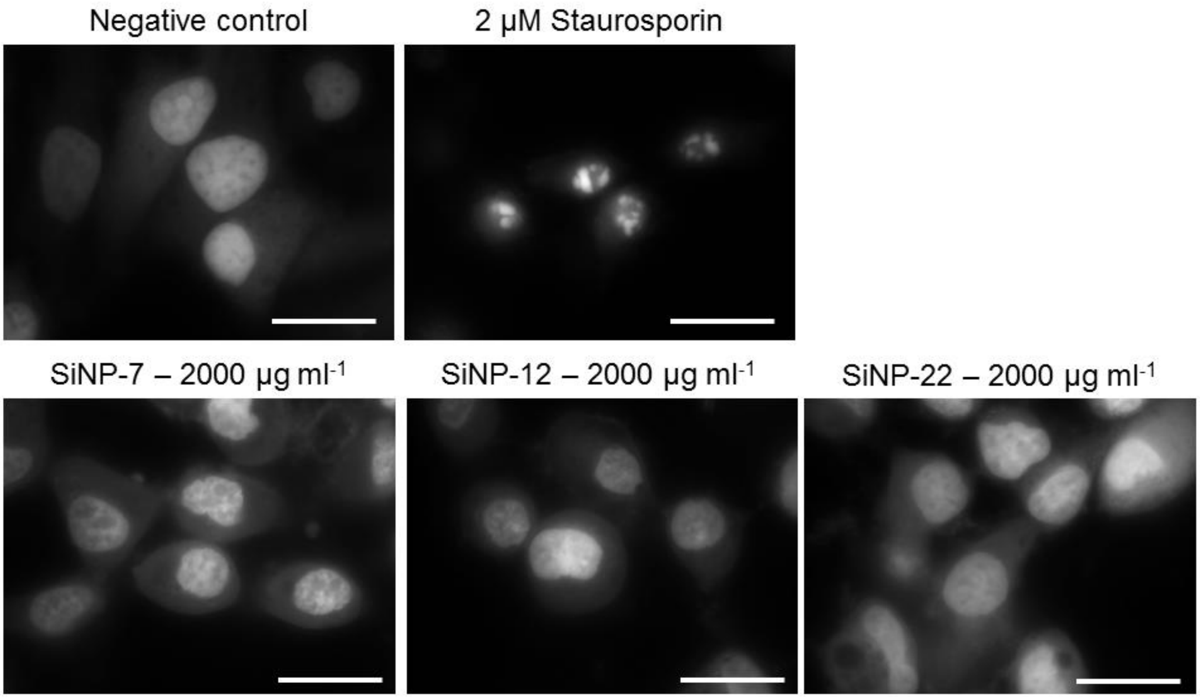

Figure S12: Analysis of chromatin condensation by Hoechst 33258 staining. Hela cells were treated with the indicated SiO_2 NP and concentrations for 2 h. Positive control: 2 µM staurosporin for 6 h (bar 10 µm). Staurosporin treatment induces a massive chromatin condensation after 6 h of exposure. No comparable chromatin condensation was found in the SiNP treated samples.

References

1. Egerton, R. F., *Electron Energy Loss Spectroscopy in the Electron Microscope.* Springer: New York, 1986. doi:10.1007/978-1-4757-5099-7

2. Brenner, D.; Mak, T. W.; Mitochondrial, cell death effectors. *Curr. Opin. Cell Biol.* **2009,** *21,* 871–877. doi:10.1016/j.ceb.2009.09.004

3. Merzlyak, E. M.; Goedhart, J.; Shcherbo, D.; Bulina, M. E.; Shcheglov, A. S.; Fradkov, A. F.; Gaintzeva, A.; Lukyanov, K. A.; Lukyanov, S.; Gadella, T. W. J.; Chudakov, D. M.; Bright, monomeric red fluorescent protein with an extended fluorescence lifetime. *Nat. Methods* **2007,** *4,* 555–557. doi:10.1038/nmeth1062
